# Genomic Differentiation, Diversity, and Genetic Structuring of *Euterpe edulis* Mart. Morphotype in Espírito Santo, Brazil

**DOI:** 10.1002/ece3.72921

**Published:** 2026-01-20

**Authors:** Jônatas Gomes Santos, Francine Alves Nogueira de Almeida, Hélio de Queiroz Boudet‐Fernandes, Pedro Henrique Dias dos Santos, Miquéias Fernandes, Suelane Costa dos Santos, Adésio Ferreira, Marcia Flores da Silva Ferreira

**Affiliations:** ^1^ Department of Agronomy Federal University of Espírito Santo Alegre Espírito Santo Brazil; ^2^ Instituto Brasileiro de Museus – IBRAM em exercício no Instituto Nacional da Mata Atlântica – INMA/MCTI Santa Teresa Espírito Santo Brazil; ^3^ Department of Plant Pathology and Crop Physiology Louisiana State University AgCenter Baton Rouge Louisiana USA

**Keywords:** conservation, functional annotation, morphological variation, SNP

## Abstract

The genomic knowledge of *Euterpe edulis* populations with morphological and genetic variations is relevant to species preservation, management, conservation, and improvement. This study aimed to identify genomic differences related to the morphological variants of *Euterpe edulis* in Espírito Santo (ES). We used 114 *Euterpe edulis* individuals, which represent different morphotypes, named: Santa Marta, Hybrid_EO (Vargem Alta); *Euterpe espiritosantensis* (Santa Teresa), Tiller (Guarapari); Possible hybrid (Fundão); characteristic of *E. edulis* (*E. edulis_*RN, *E. edulis_*MI, *E. edulis_*GUA, and *E. edulis_*ALE). The study also included 35 individuals from divergent genetic groups in natural populations from the southeast, north, south, and center‐west Brazil (Federal District). After filtering, 5319 SNPs were used in the genetic diversity and structure. Groups of SNPs differentiating morphotypes were identified and subjected to functional annotation analysis using as reference the *Elaeis guineensis* genome. Orthology and abundance analysis by Gene Ontology were made. Individuals of Hybrid_EO displayed the highest genetic diversity (*He* = 0.23). The other morphotypes displayed *He* between 0.107 (*E. espiritosantensis*) and 0.197 (*E. edulis_*RN). The inbreeding coefficient (*Fis*) varied between 0.024 (*E. edulis_*RN) and 0.255 (Hybrid_EO). Genetic variation was 42.99% between morphotypes and 57.00% within morphotypes. A *k* = 4 with individuals with slight admixture was identified. The morphotype *E. espiritosantensis* was more differentiated. The possible hybrid and progenitor morphotype grouped with *E. oleraceae* and *E. precatoria* species in the phylogenetic tree. Alignment of the 1627 SNPs in the *Elaeis guinensis* genome highlighted 767 aligned sequences in genic regions, of which 195 were in coding regions. The annotation of SNPs for the Hybrid_EO and Tiller morphotypes demonstrated different alleles for genes related to response to stress and environmental stimuli. The identification of common GOs indicates a common genetic background, while the presence of SNPs with differentiating genotypes suggests specific adaptations to different environmental conditions.

## Graphical abstract

This study investigates genomic differences among distinct morphotypes of *Euterpe edulis* in Espírito Santo (ES), Brazil, using a dataset of 5319 high‐quality SNPs.

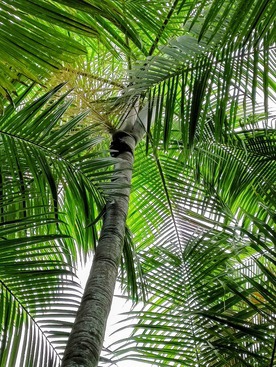

## 1 Introduction

*Euterpe edulis* Mart. (Areaceae) is a palm tree widely distributed in the Atlantic Forest (Vianna 2020) that has ecological, economic, and social importance (Barroso et al. 2010; Galetti et al. 1999; Genini et al. 2009). It has potential for the bioeconomy and sustainable development through its fruits, which produce a pulp similar to açaí, the pulp of the fruits produced by *Euterpe oleraceae* Mart. in the Amazon. With this use, juçara fruits have gained market space (Vianna 2020). The natural populations of this palm tree present high genetic diversity and different genetic groups (Mengarda et al. 2022; De Moraes et al. 2020; Pereira et al. 2022). This information is relevant to guide conservation actions, sustainable management, and species improvement (De Carvalho et al. 2022; De Moraes et al. 2020).

Two genetic groups for *E. edulis* have been identified throughout the Brazilian Atlantic Forest, geographically dividing this biome into north and south, with an intermediate zone in the Brazilian southeast region (Pereira et al. 2022; de Almeida et al. 2024). The state of Espírito Santo (Brazil) presents a high natural diversity of *E. edulis*, both genetically and morphologically (Carvalho et al. 2020; Coelho et al. 2020; Fernandes 1989; Pereira et al. 2022; Wendt et al. 2011). For example, there is the morphological description of *Euterpe espiritosantensis* H.Q.B.Fern, which was initially considered a new species and, after a taxonomic review, was classified as a heterotypic synonym of *E. edulis* (Fernandes 1989; Wendt et al. 2011). Scientific research also reported tillering individuals, with variations in fruit size, stem diameter, meristem color, and number of rachillae (Fernandes 1989; Mantovani and Morellato 2000; Wendt et al. 2011). Additionally, there are reports of planting supposedly hybrid individuals to produce palm hearts on a commercial scale (Bovi et al. 1987). Together, this information suggests the existence of intra and interspecific hybrids, a hypothesis that requires an in‐depth investigation.

The genomic study of these morphological variants can explain the genetic causes of phenotypic differentiation and genetic improvement studies to identify genes of agronomic interest related to morphotypes (Cerqueira et al. 2022; Young et al. 1996). Studies of genetic diversity and structure of natural populations for *E. edulis* are generally carried out using the same set of highly polymorphic microsatellite *loci* (Carvalho et al. 2017; Cerqueira et al. 2022; Coelho et al. 2020; Gaiotto 2003; Mengarda et al. 2022; De Moraes et al. 2020; Pereira et al. 2022; de Almeida et al. 2024). The use of *Single Nucleotide Polymorphism* (SNPs) is widely spread in the genome and the SNP obtained by DArTseq methodology, preferentially occurring in genic regions (Dwiningsih et al. 2020; Sansaloni et al. 2011), opens up possibilities besides genetic diversity studies, to generate information associated with the genomic differentiation of groups, enabling the annotation of SNP regions that differentiate between groups (Ding et al. 2023; Zhang and Zhang 2022). However, the SNPs which differentiate *E. edulis* morphotypes could be related to environmental responses due to neutral evolutionary processes, founder effects or genetic drift; SNPs in coding regions can also be an indicator of putative functional effects on transcription or phenotype. These types of data would add to the knowledge for a more comprehensive understanding of genetic adaptation of *E. edulis* .

In this study, genomic SNPs differentiated the morphotypes of *Euterpe edulis* occurring in Espírito Santo, using analyses of diversity, genetic structure, phylogeny, and functional annotation of SNPs, aiming to increase knowledge of the genetic bases related to the phenotypic variation of the species. The results of this study may impact actions in conservation and improvement programs for the species.

## 2 Materials and Methods

### 2.1 Plant Material

Of the 114 samples of *Euterpe edulis* used, 79 were from individuals collected in nine locations in fragments of the Atlantic Forest in Espírito Santo (ES), Brazil. These locations were determined as sampling points because they presented records of individuals that differed in morphological attributes (Figure S1). The remaining 35 individuals derived from other populations in Brazil. Five individuals derived from the southeast (Rio de Janeiro—RJ); 10 individuals from the north (Alagoas—AL); nine individuals from the south (Rio Grande do Sul—RS); and 10 individuals from the center‐west (Distrito Federal—DF). These populations represent distinct genetic groups in Brazil and from the typical populations of *E. edulis* collected in Espírito Santo (Pereira et al. 2022; de Almeida et al. 2024). In this work, the morphological variants will be called morphotypes (Table 1).

**TABLE 1:** Description of the populations studied in terms of location, identification (Id), morphotype, number of individuals collected (*N*) in each location, latitude, and longitude.

Location	Municipality	Id	Morphotype	*N*	Latitude	Longitude	Altitude (m)
Acai Juçara, Bonalotti—Private Property	Rio Novo do Sul, ES	*E. edulis_*RN	*E. edulis*	11	−20.807598	−40.934519	470
Private propriety	Guarapari, ES	Tiller	Tiller	8	−20.46300	−40.56493	41
Augusto Ruschi Biological Station—INMA	Santa Teresa, ES	*E. espiritosantensis*	*E. espiritosantensis*	9	−19.965455	−40.540530	900
Santa Lucia Biological Reserve	Santa Teresa, ES	*E. espiritosantensis*	*E. espiritosantensis*	3	−19.65512	−40.540134	663
Goiapaba Açu Park	Fundão ES	Possible hybrid	Possible hybrid (*E. edulis × E. espiritosantensis*)	7	−20.46305	−40.56493	791
Taquarussu—Private Property	Vargem Alta ES	Híbrid_EO	Hybrid *E. edulis ×* *E. oleracea*	6	−20.33564	−40.58296	707
Castelinho—Private Property—	Vargem Alta ES	Santa Marta	Santa Marta	15	−20.31214	−40.59268	869
San Rafael—Private Property	Mimoso do Sul ES	*E. edulis_*MI	*E. edulis*	10	−21.062313	−41.363282	670
Private propriety	Guaçui ES	*E. edulis_*GUA	*E. edulis*	5	−20.808555	−41.623972	687
Private propriety	Alegre ES	*E. edulis_*ALE	*E. edulis*	5	−20.807722	−41.515805	695
Private propriety	Paraty, RJ	*E. edulis_*RJ	*E. edulis*	5	−23.213713	−44.793159	206
IBGE Ecological Reserve	Brasilia, DF	*E. edulis_*DF	*E. edulis*	10	−15.948141	−47.878507	1112
Itapeva State Park	Torres, RS	*E. edulis_*RS	*E. edulis*	9	−29.39918	−49.75948	266
Murici Ecological Station	Murici, AL	*E. edulis_*AL	*E. edulis*	10	−9.25597	−35.83972	408

*Note:* The acronyms used to name the morphotypes follow the pattern of the name of the morphotype and place where they are found. The populations of the characteristic morphotype have only the name of the place and the state. *E. edulis_*RN—Rio Novo do Sul, ES; Tiller—Guarapari, ES; *E. espiritosantensis*, Santa Teresa, ES; Possible hybrid, Fundão, ES; Hybrid_EO—Vargem Alta, ES; Santa Marta—Vargem Alta, ES; *E. edulis_*MI—Mimoso do Sul, ES; *E. edulis_*GUA—Guaçuí, ES; *E. edulis_*ALE—Alegre, ES; *E. edulis_*RJ—Rio de Janeiro; *E. edulis_*DF—Federal District: *E. edulis_*RS—Rio Grande do Sul; *E. edulis_*AL—Alagoas.

**FIGURE 1:**
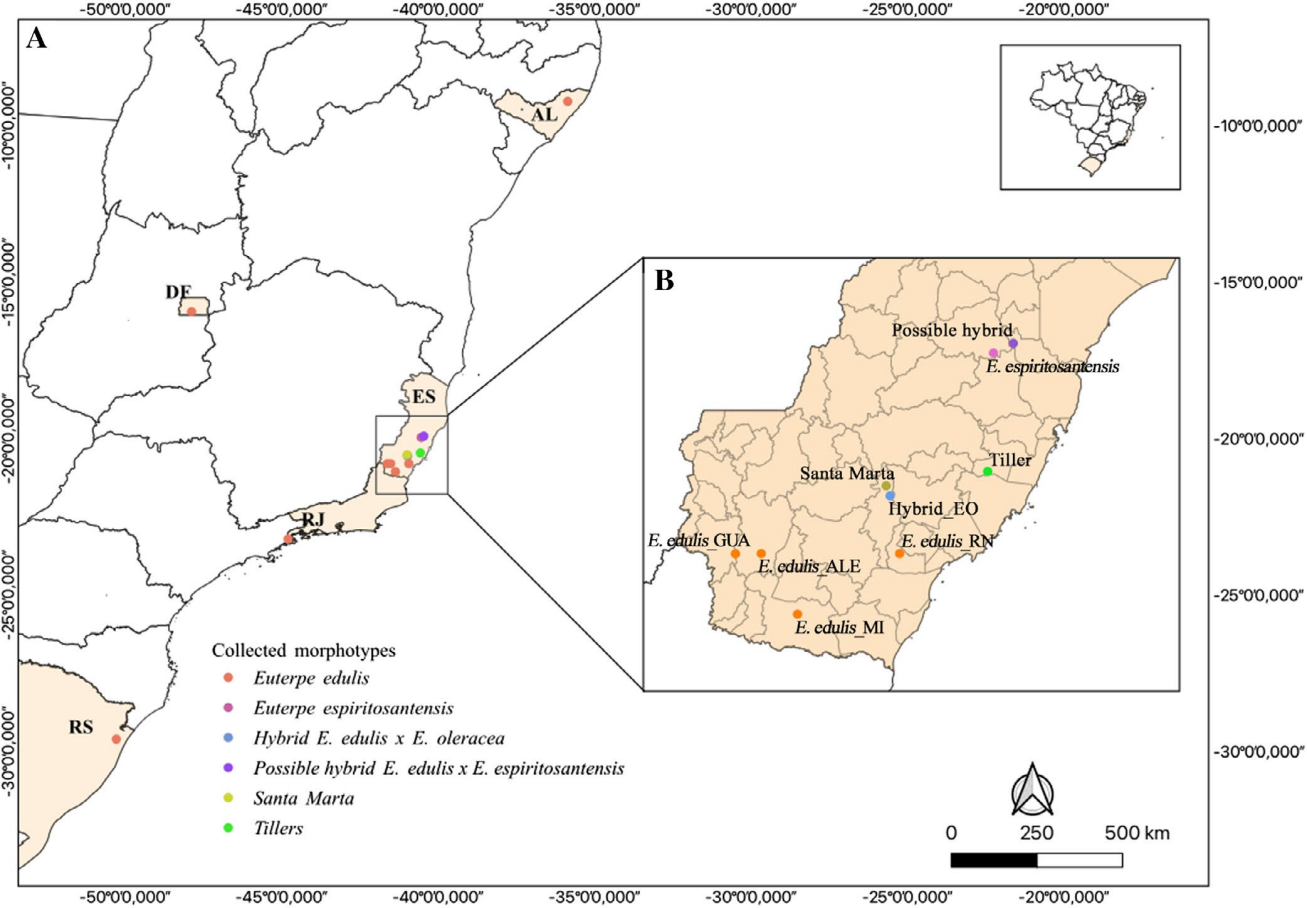
Sampling sites of *Euterpe edulis* morphotypes. (A) Sampling locations of the *E. edulis* characteristic morphotypes in Brazil. (B) Municipalities in Espírito Santo where the 79 individuals of *E. edulis* used in this work were collected. The small map in the top right corner displays the geographic map of Brazil.

The genomic data of individuals collected in ES were compared to 35 individuals collected in natural Brazilian populations, representing different genetic groups (Pereira et al. 2022). Pereira et al. (2022) described the collection of these samples, and the collections were performed in the Federal District, Alagoas, Rio de Janeiro, and Rio Grande do Sul. Samples were harvested under the authorization issued by the Biodiversity Authorization and Information System (SISBIO) number 87764‐2 for activities with scientific purposes. The collection sites in ES were characterized with the aid of the Global Positioning System (GPS) (Figure 1).

### 2.2 Obtaining Genomic DNA and Sequencing

Leaves and stems of adult individuals were collected and stored in paper bags containing silica gel to preserve DNA (Carvalho et al. 2020). After collection and transportation, the material was frozen at −80°C and lyophilized for 72 h. Genomic DNA extraction was performed using the cetyltrimethylammonium bromide (CTAB) method of Doyle and Doyle (1990), with modifications (Carvalho et al. 2020; Ferreira and Grattapaglia 1998). The protocol included three steps of protein removal with chloroform: alcohol isoamyl acetate in a 24:1 ratio and DNA precipitation without ammonium acetate. The purified DNA was then quantified in the NanoDrop. Samples that presented a 260/280 nm ratio between 1.8 and 2.0 were genotyped. Genomic DNA from the samples was sent to the Genetic Analysis Service for Agriculture Laboratory (SAGA in Spanish, Texcoco—Mexico). The DNA complexity reduction procedure was performed using two restriction enzymes *MseI* and *Hpall* (Sansaloni et al. 2011). After digestion with restriction enzymes, the DNA fragments were connected to adapters, one adapter for sample identification and another specific for recognition by the Illumina sequencer Novaseq 6000 (Sansaloni et al. 2011).

### 2.3 SNP Processing and Quality

The SNPs were filtered using the dartR package (Gruber et al. 2018) in the R software (R Core Team 2021). This package classifies as ‘0’ homozygous for the reference allele, ‘1’ heterozygous allele for the SNP, and ‘2’ being homozygous for the alternative allele. Three individuals were excluded, as they had more than 20% missing data. The following parameters were used for filtering: reproducibility > 0.99, call rate > 0.95, minor allele frequency (MAF) > 0.05, and secondary SNPs. After filtering, 5319 of 93,682 SNPs were kept for subsequent analyses of the 79 individuals from Espírito Santo. Five of the 114 individuals presented more than 20% missing data and were excluded. After filtering, 2982 of 93,682 SNPs were kept for subsequent analyses of the 109 individuals.

### 2.4 Analysis of Genetic Diversity and Structuring

With the filtered SNPs, the “gl.smearplot” function from the dartR package (Gruber et al. 2018) demonstrated the individuals' genomic profiles. Thus, a heatmap of the possible states of each *locus* per individual was obtained, which could be one of the two homozygous or heterozygous per *locus*. Roger's distance (1972) and UPGMA (Unweighted Arithmetic Average of Pairwise Clusters) grouping method divided the individuals into groups using the “poppr” package (Kamvar et al. 2014). The SNP grouping was performed using the “distfun” function of the heatmap package, which uses Euclidean distance. Two analyses were performed, one using data from all individuals and the other using only the 79 individuals collected from Espírito Santo. The genetic diversity parameters, expected heterozygosity (*He*), observed heterozygosity (*Ho*), and inbreeding coefficient (*Fis*) of the populations were estimated using the packages “diversity” (Keenan et al. 2013), “hierfstat” (Goudet 2005), and “poppr” (Kamvar et al. 2014) based on Weir and Cockerham (1984), which takes into account the finite size of the samples and populations. Molecular analysis of variance (AMOVA) was performed using the “poppr” package (Kamvar et al. 2014) in the R software (R Core Team 2021) to verify the variation within and between populations and morphotypes.

The differentiation among the populations (*Fst*) was estimated using Nei's genetic distance (Nei and Li 1979) and UPGMA clustering with the “hierfstat” package in R (Goudet 2005). The “boot.ppfst” function was used with parameters nboot = 1000 and quant = c (0.025, 0.975) to obtain confidence intervals for *Fst* values and test their significance. The population structure was analyzed using the LEA V2.8.0 package (Frichot and François 2015) that uses Principal Component Analysis (PCA) and mixture analysis (Patterson et al. 2006; Pritchard et al. 2000) in the RStudio software (R Core Team 2021).

To evaluate the relationship between genetic structure and geographic distribution, we performed an additional spatially explicit ancestry analysis using the TESS3 algorithm implemented in the *tess3r* R package (Caye et al. 2016). This approach integrates individual genotypes with geographic coordinates to infer ancestry coefficients while accounting for spatial autocorrelation. The analysis was conducted using the same filtered SNP dataset applied in the population structure analyses and the geographic coordinates obtained at the sampling sites. Ancestry coefficients were estimated for *K* values ranging from 4 to 6, based on cross‐validation scores and biological interpretability. Spatial interpolation of ancestry coefficients was performed using kriging, allowing visualization of the spatial distribution of genetic clusters across Espírito Santo. The resulting maps are presented in Figure S3.

### 2.5 Phylogenetic Analysis

Genotypic data were analyzed using the dartR package in R software. Monomorphic loci were filtered, followed by a reproducibility filter with a threshold = 0.99. Subsequently, the filter.depth function was used to filter loci with reading depth less than five or greater than 50 (lower = 5, upper = 50) and discarded. The call rate was performed with a threshold = 0.95, the maf with threshold = 0.02, and secondary SNPs were filtered. After filtering, the file was converted to fasta format using the gl2fasta function.

Maximum likelihood phylogenies were inferred by IQ‐TREE, using the TVMe+R3 model according to the Bayesian Information Criterion (BIC). Five hundred bootstraps and 1000 Shimodaira–Hasegawa–like approximate replications. The likelihood ratio test and the minimum correlation coefficient were set to 0.90. The parameter “‐spp” indicated that each partition could have an evolution rate.

### 2.6 Functional Annotation of SNP Groups

**FIGURE 2:**
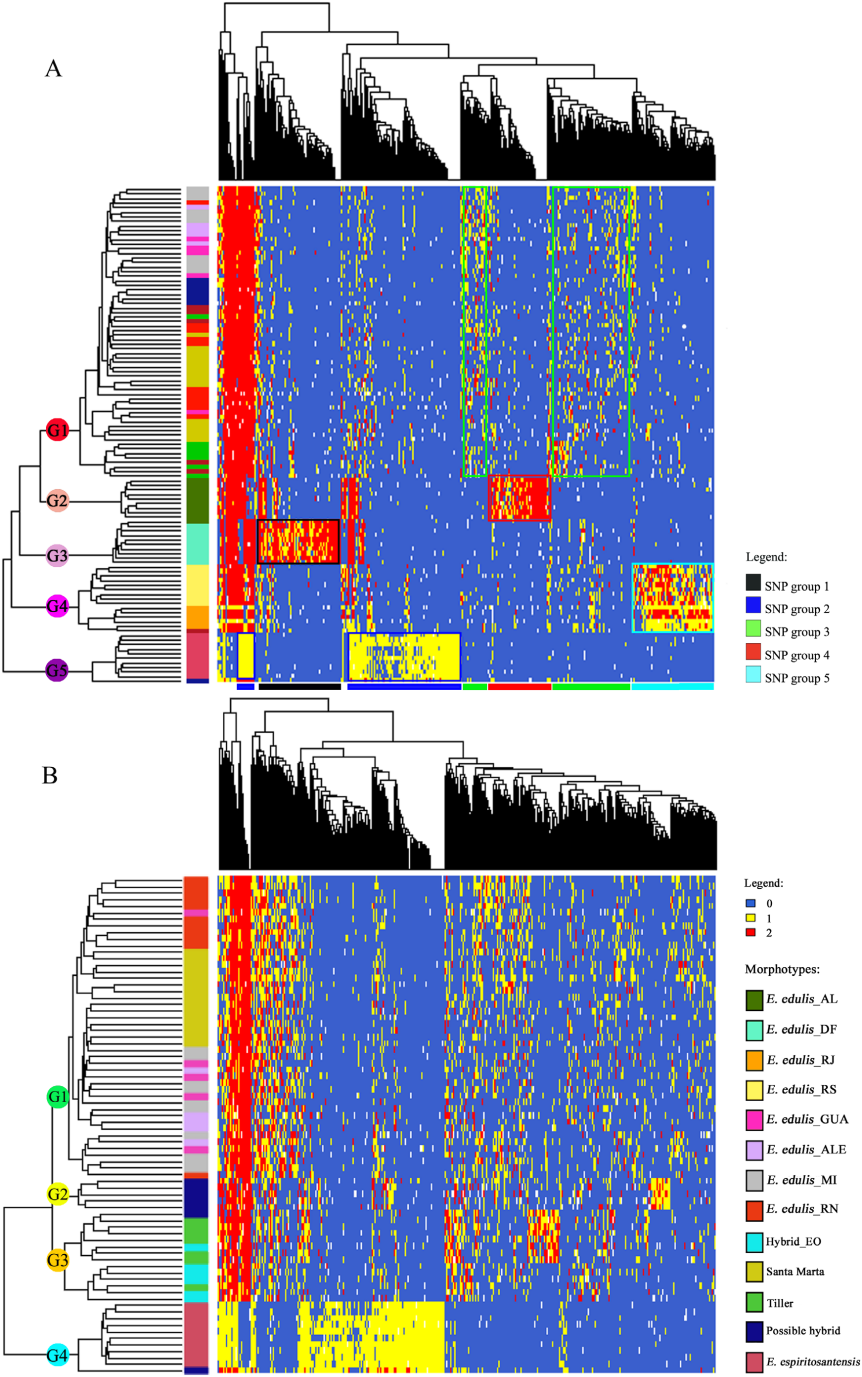
(A) Genotypic variability of the analyzed populations. Genotypic profile showing the allelic diversity found in the different populations analyzed considering reference populations in DF, AL, RJ, and RS (2982 SNPs). Identification of SNP groups with differential genotypes in genetic groups of individuals formed. The lower bars indicate the groups of SNPs chosen in each genetic group. (B) Groupings based on the genomic profile of 76 individuals of *E. edulis* from ES (row) with 5319 SNPs (column) distributed in the genome of the species; G1, G2, G3, and G4 represent the groups of morphotypes formed based on the Euclidean distance. The colors in each row represent the genotypes of each individual for each of the SNPs, in which blue—represents the homozygote for the reference allele, red—is the homozygote for the alternative allele, and yellow—is the heterozygote.

From the groups of individuals obtained based on the genomic profile of the 109 individuals, we could select the SNPs that differentiated morphotypes using the R Software (R Core Team 2021). The order of the SNPs was obtained using the “*hv*” function of the heatmap package. Five groups of SNPs that differentiated the genetic groups of individuals were selected (Figure 2a). The annotation was conducted only for the SNPs differentiating each genetic group.

For the functional annotation of the SNPs, we used Google Colaboratory and a Python script (Supporting Information), with the Pandas, Seaborn and Biopython libraries (Cock et al. 2009). The genome reference sequence of *Elaeis guineensis* (GCF_000442705.1), including structural annotation (GFF3), mRNA, and protein, was obtained from NCBI (O'Leary et al. 2016). It was used for annotation, with objective study SNPs only conserved genes between species as studied by de Medeiros Cardozo et al. (2025). The sequences of the SNPs in the regions of interest were extracted using the Biopython library and formatted in a FASTA file. A local alignment was performed using BLASTn (version 2.12.0+) with the *e*‐value (1 < e^−5^) and output format 6. The relationship between genes, mRNA, and proteins was extracted from the GFF3 file, specifically in the CDS feature lines. The proteins corresponding to the SNPs regions were functionally annotated using InterProScan 5 (Jones et al. 2014) in Galaxy.eu version 5.59–91.0 + galaxy3 (Afgan et al. 2022), with the PFAM (El‐Gebali et al. 2019) and PANTHER (Thomas et al. 2022) databases. One hundred fifty nonredundant SNPs were annotated with GO terms (Aleksander et al. 2023). The GO terms were consolidated using the GO Slim tool available on the AgBase platform (McCarthy et al. 2007). Graphs grouped by aspect and group were generated using the Seaborn library, and the annotations were integrated.

## 3 Results

### 3.1 Genomic Profile

The clustering analysis reveals five genetic groups for the 109 individuals (Figure 2A) and four groups for individuals collected in Espírito Santo (Figure 2B). In the analysis of all individuals (2982 SNPs), individuals from AL (*E. edulis_*AL), DF (*E. edulis_*DF), and *E. espiritosantensis* formed individual groups (G2, G3, G5, respectively); individuals from RJ (*E. edulis_*RJ) and RS (*E. edulis_*RS) grouped (G4), as well as all other individuals collected in ES (G1). In the analysis of the 76 individuals from ES (5319 SNPs) (Figure 2B): Group G1 grouped *E. edulis* and the Santa Marta morphotype; G2 grouped the *E. edulis* individuals collected in Fundão, ES; G3 included tillering individuals and hybrid individuals between *E. edulis* and *E. oleracea* (Hybrid_EO). G4 grouped individuals of *E. espiritosantensis* and one individual possible hybrid collected in Fundão, ES (Figure 2B).

The *E. espiritosantensis* presented a distinct genomic profile (Figure 2A,B), generally presenting only two of the three possible genotypes per SNP, with one of the homozygotes for the alternative allele being practically absent, thus a large number of SNPs with a homozygous genotype for the reference allele (blue) and the rest of the SNPs in heterozygosis (yellow) (Figure 2). This morphotype presented the most significant genetic distance to the other groups, including the highly divergent populations used as a reference (Pereira et al. 2022).

### 3.2 Genetic Diversity of *Euterpe* Populations in Espírito Santo

Regarding estimates of the genetic diversity of the individuals from Espírito Santo (Table 2), the average values of *Ho* and *He* were 0.154 and 0.192, respectively. The *E. espiritosantensis* displayed the lowest Ho *Ho* and He *He* (0.093 and 0.107, respectively). The individuals Hybrid_EO displayed the highest *He* (0.233) and F *Fis* (0.257), followed by the tillering morphotype (*He* = 0.193; *Fis* = 0.197). The population managed for fruit production (*E. edulis_*RN) displayed the lowest *Fis* (0.024). Considering the total sample of 109 individuals (Table 2), the *E. espiritosantensis* had the lowest genetic diversity, behind only the individuals from DF and AL, the least diverse.

**TABLE 2:** Diversity parameters of *Euterpe edulis* for different morphotypes sampled in Espírito Santo considering the analysis of 5319 SNPs. Observed heterozygosity (*Ho*), expected heterozygosity (*He*), and inbreeding coefficient (*Fis*), and significance values.

Groups	Morphotype	Location	*Ho*	*He*	*Fis*	> 95%	< 95%	Sig.
G1	*E. edulis_*ALE	Alegre	0.151	0.187	0.135	0.172	0.211	*
G1	*E. edulis_*GUA	Guaçuí	0.160	0.192	0.114	0.148	0.184	*
G1	*E. edulis_*MI	Mimoso do Sul	0.152	0.185	0.143	0.166	0.193	*
G1	*E. edulis_*RN	Rio Novo do Sul	0.191	0.197	0.024	0.015	0.040	*
G1	Santa Marta	Vargem Alta	0.174	0.196	0.092	0.105	0.127	*
G2	Possible hybrid	Fundão	0.154	0.187	0.125	0.158	0.189	*
G3	Hybrid_EO	Vargem Alta	0.160	0.233	0.257	0.289	0.333	*
G3	Tiller	Guarapari	0.147	0.193	0.197	0.220	0.255	*
G4	*E. espiritosantensis*	Santa Teresa	0.093	0.107	0.100	0.108	0.150	*
	Average	—	0.154	0.192	0.125	0.158	0.189	

*Note:* *Ho* = number of homozygotes/number of individuals; *He* = 1 − (*p* ^2^ + *q* ^2^); Coefficient of inbreeding (FIS) = 1 − (mean(*Ho*)/mean(u*He*)); estimates of inbreeding in the population are derived using the Weir and Cockerham estimator. Estimates with a 95% confidence interval including zero are considered not significantly different from zero (ns); however, if zero is not contained in the interval, inbreeding is considered significantly different from zero (*).

Considering the collection sites of individuals from Espírito Santo was estimated 41.63% genetic variation between sites and 58.36% within sites (Table 3). Defining the morphotypes as a hierarchy 42.99% of the genetic variation was identified between morphotypes and 57.00% within morphotypes (Table 3). The analysis between groups revealed that 47.63% of the genetic variation was within groups and 52.36% among them (Table 3). The comparison between Espírito Santo's data and Brazil's populations detected a higher variation among the populations (66.71%). However, defining the morphotypes as hierarchy, the most significant variation occurred within morphotypes (73.87%) (Table S1).

**TABLE 3:** Molecular Analysis of Variance (AMOVA) for different morphotypes of *Euterpe edulis* considering 5319 SNPs with varying levels of hierarchy, the first being between and within populations, the second between and within morphotypes, and the third between groups formed based on the genomic profile.

Source of variation	Degrees of freedom	Sum of squares	Mean squares	Sigma	% Variation	Phi
Between locations	8	31,323.08	3915.38	403.61	41.63	
Inside the locations	67	37,900.66	565.68	565.68	58.36	
Total	75	69,223.74	969.29	480.48	100.00	0.41
Between morphotypes	5	28,178.76	5635.75	442.31	42.99	
Within the morphotypes	70	41,044.97	586.35	586.35	57.00	
Total	75	69,223.74	922.98	1028.67	100.00	0.42
Between groups	3	25,882.19	8627.39	547.51	47.63	
Within groups	72	43,341.54	601.96	601.96	52.36	0.47
Total	75	69,223.74	922.98	1057.84	100.00	

**FIGURE 3:**
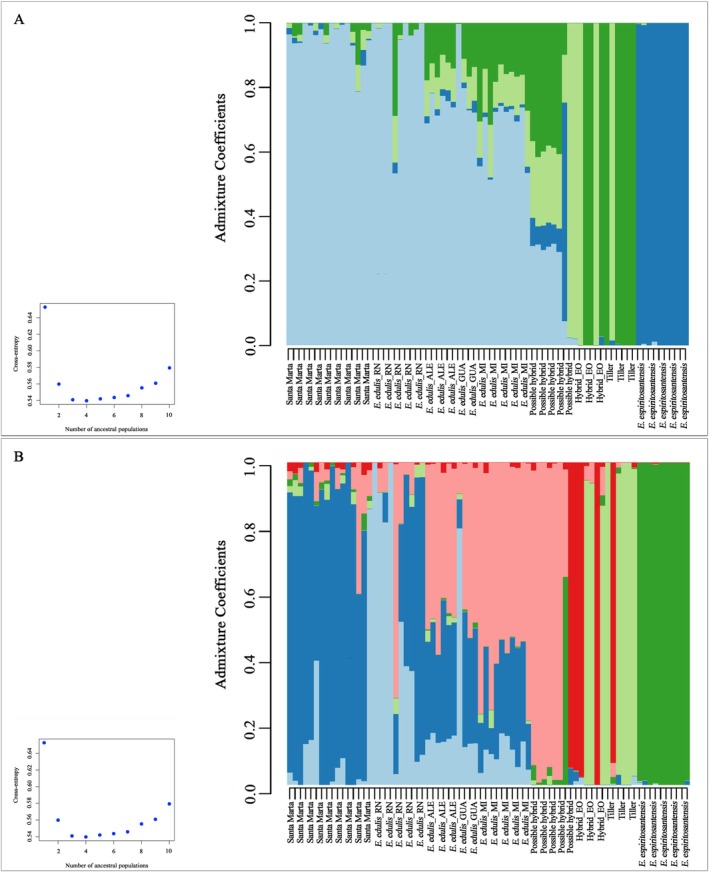
Population structure analysis of 76 *Euterpe edulis* individuals collected in Espírito Santo, obtained through the LEA package and based on 5319 SNPs. (A) The value of *K* = 4 was the most statistically significant and best explained the distribution of morphotypes according to the gene cluster. (B) Contributions of each genetic group in each morphotype if *K* = 6 were selected according to the number of morphotypes evaluated.

The structuring analysis considering the individuals from ES revealed a statistically significant *K* value of four genetic groups (*K* = 4) (Figure 3A). The *E. espiritosantensis* morphotype displayed being structured (Figure 3A). The tillering individuals and Hybrid_EO were positioned in two high‐structure genetic groups. The individuals from Santa Marta were structured. However, the predominant genetic group is shared with the other populations of individuals characteristic of *E. edulis* found in ES and a low proportion of genetic groups of *E. espiritosantensis*, tiller, and Hybrid_EO. The possible hybrid individuals from Fundão, ES had the most significant admixture and included all other genetic groups, with individuals with high admixture (Figure 3A). The typical individuals of *E. edulis* collected on private properties in Alegre, Guaçuí, and Mimoso do Sul also showed an admixture of genetic groups (Figure 3A).

Considering the value of *K* = 6, the individuals of *E. espiritosantensis* remain highly structured to the others (Figure 3B). The tillering morphotype and the Hybrid_EO still shared two genetic groups with high structure within the individuals. It was now possible to notice a prevailing genetic group among the individuals of Santa Marta, differentiating them from the typical individuals of the *E. edulis* morphotype. The typical individuals of the *E. edulis* morphotype displayed average structure, with individuals containing varied proportions of different genetic groups. The individuals of the possible hybrid morphotype remained well structured, with a high proportion of the same genetic group, except for one individual that showed a proportion of the genetic group present in *E. espiritosantensis*. However, when observing the individuals of this morphotype at *K* = 4 (Figure 3A), they appear to be admixed and display a greater proportion of an ancestral genetic cluster shared with the other populations collected in Espírito Santo.

The spatially explicit ancestry analysis revealed a clear correspondence between genetic structure and geographic distribution of the morphotypes sampled in Espírito Santo (Figure S3). For *K* = 4, the genetic clusters identified by TESS3 exhibited coherent spatial patterns, with *Euterpe espiritosantensis* forming a geographically restricted and highly structured cluster in Santa Teresa, whereas individuals of *E. edulis* from different municipalities showed broader spatial overlap and higher admixture. The tillering morphotype and the Hybrid_EO individuals were spatially associated and shared similar ancestry profiles, reinforcing their genetic proximity. The possible hybrid population from Fundão displayed a spatially heterogeneous pattern, consistent with the high admixture observed in nonspatial structure analyses. Increasing *K* to 5 and 6 resulted in further subdivision of genetic components; however, these additional clusters showed substantial spatial overlap and did not correspond to clearly distinct geographic or biological units.

**FIGURE 4:**
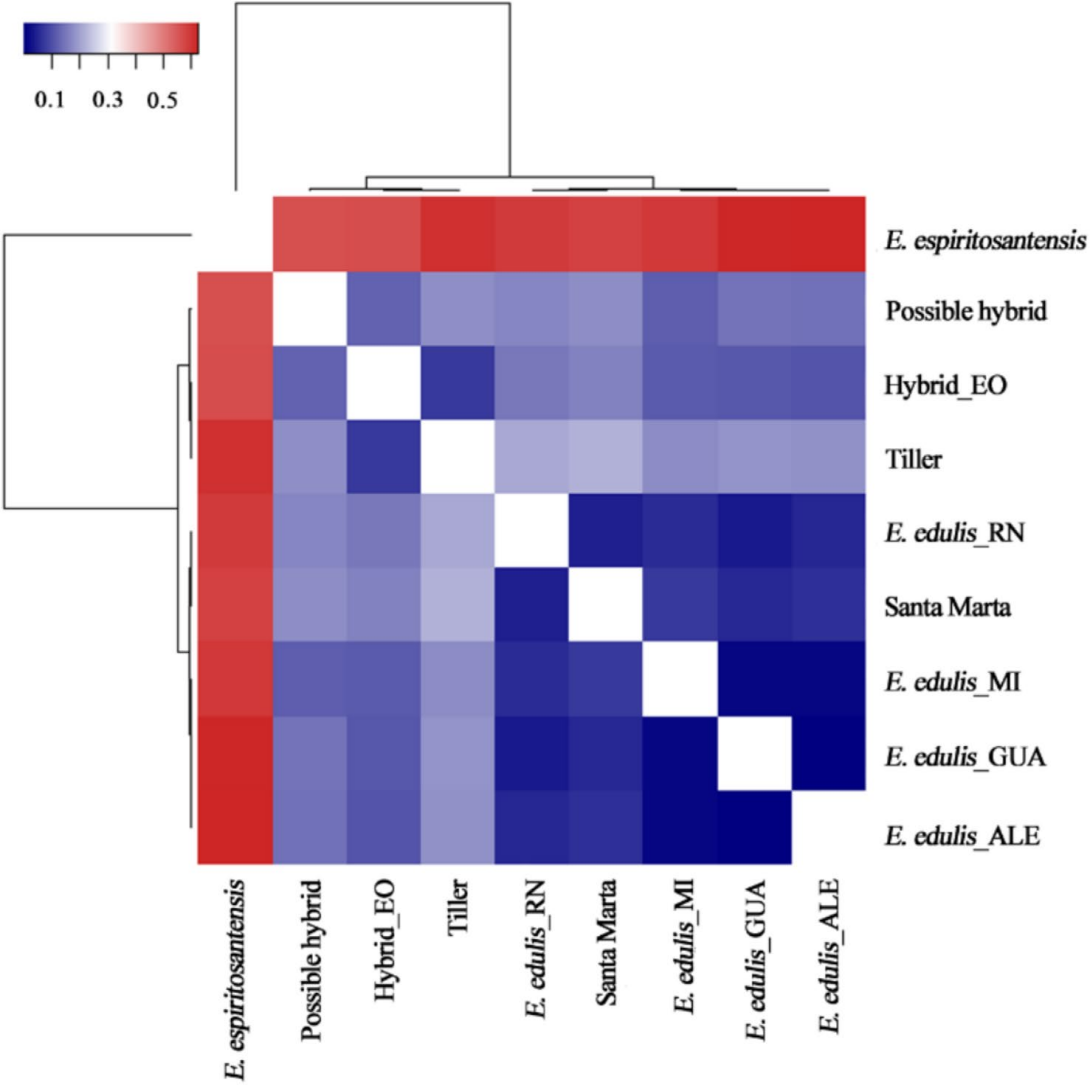
Genetic differentiation (*Fst*) between the studied morphotypes. In red, the average differentiation between the morphotypes is higher. In blue, the average differentiation is lower.

The genetic differentiation based on *Fst* corroborated the results of the structuring analysis. The *Fst* was much higher among the *E. espiritosantensis* individuals than any other morphotype (values above 0.5) (Figure 4; Table S3). The second most significant differentiation was of individuals with tillering, followed by possible hybrid. The Santa Marta morphotypes and characteristics of *E. edulis* presented lower *Fst* values (Table S2).

### 3.3 Phylogenetic Analysis

**FIGURE 5:**
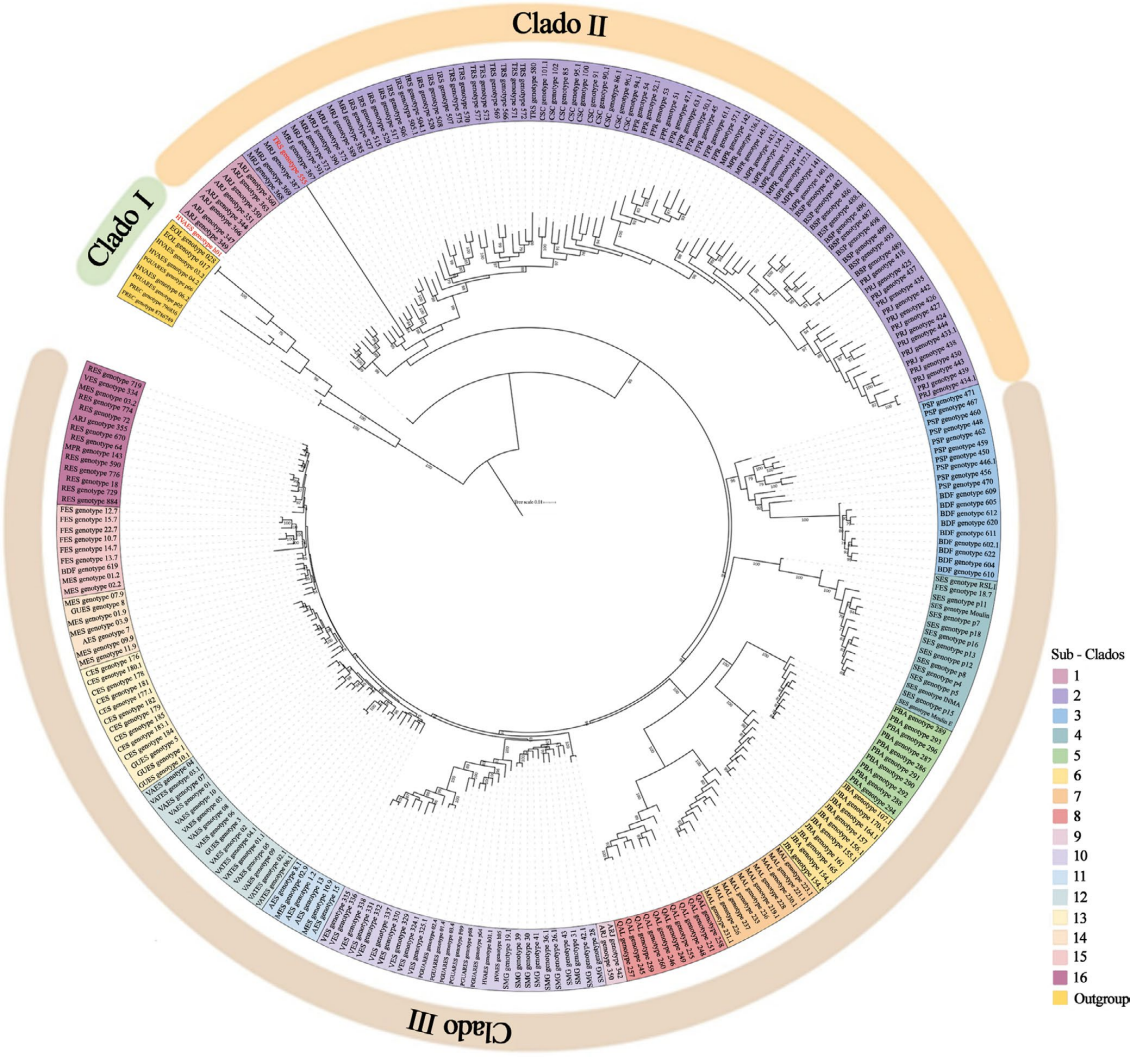
Phylogeny of *Euterpe edulis* based on SNPs. The phylogeny was constructed with 4811 SNPs and 258 individuals from different populations in Brazil, forming three clades.

The phylogenetic reconstruction generated three large clades: one included the species used as an outgroup and individuals profiled Hybrid_EO (possible hybrids between *E. edulis* × *E. oleraceae*) collected in Espírito Santo. The other two large clades separated into two populations. The first comprised individuals from the southeast (ARJ, MRJ, PRJ, and BSP) and south of Brazil. The second included individuals from the northeast (PBA, JBA, QAL, and MAL), including the morphotypes found in Espírito Santo and a population from Minas Gerais. The morphotype *E. espiritosantensis* was evolutionarily distant from the other Espírito Santo populations, approaching the Federal District population (Figure 5). In addition, this morphotype was grouped with the populations of Bahia (PBA and JBA) and Alagoas (QAL and MAL). The *E. espiritosantensis* populations of Federal District, Alagoas, and Bahia presented high node support values (bootstrap between 95 and 100%), showing the reliability of the groupings formed.

### 3.4 Functional Annotation of SNPs Differentiating Morphotypes in ES

Of the 2984 SNPs analyzed in 109 individuals, 2706 SNPs presented genotypes differentiating the genetic groups of individuals. These SNPs were grouped into five groups (Figure 2A). Group 1–410 SNPs with differential genotypes for *E. edulis* individuals collected in DF (*E. edulis_*DF). Group 2–986 SNPs with differentiating genotypes for *E. espiritosantensis* individuals. Group 3–300 SNPs with differentiating genotypes for characteristic *E. edulis* individuals collected in ES, including the Hybrid_EO and Santa Marta. Group 4, composed of 460 SNPs with genotypes exclusive to individuals collected in AL, and Group 5, composed of *E. edulis* collected in RJ (*E. edulis_*RJ) and RS (*E. edulis_*RS), presented 550 SNPs (Table S1).

**FIGURE 6:**
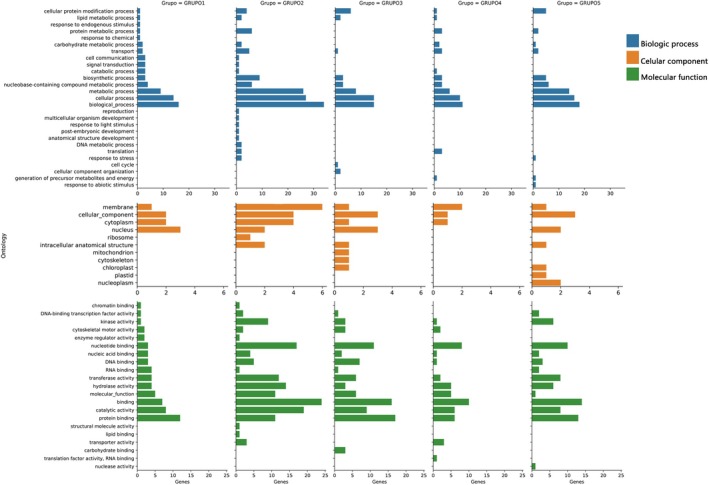
Molecular enrichment functions of the annotated differentiating SNPs groups highlighting the biological processes that are related. Group 1, SNPs with differential genotypes for *E. edulis* individuals from DF; Group 2, differential SNPs for *E. espiritosantensis* individuals; Group 3, SNPs with genotypes characteristic of ES; Group 4, SNPs differentiating individuals of AL; Group 5, SNPs differentiating individuals of *E. edulis* from RJ and RS.

Alignment of the 2706 differentiating SNPs in the *Elaeis guineensis* genome displayed 1881 alignments, of which only 250 were nonredundant and aligned to the mRNA sequence. The alignment of *Euterpe edulis* sequences on the GFF file of *Elaeis guineensis* reveals conserved genes as shown by de Medeiros Cardozo et al. (2025). Among the 250 nonredundant alignments, 154 sequences were annotated with Gene Ontology (GO) (https://www.geneontology.org/) which are included in the five SNP groups identified. The functional annotation demonstrated that this small set of SNPs was related to three main categories, as standard provided by Gene Ontology (GO) (Figure 6): biological processes, cellular components, and molecular function. Group 1 of SNPs included SNP genotypes exclusive to *E. edulis* individuals (DF), presented 15 GOs related to the biological process, four to the cellular component, and 15 to the molecular function.

Group 2 of SNPs, composed of SNPs with genotypes differentiating *E. espiritosantensis* individuals, presented the highest number of GOs related to biological process (24) and molecular function (18) and only six to the cellular component. Among the GOs found for biological processes in this region, some associated with reproduction, cell death, development, response to stress, response to light stimuli, and structural anatomical development, exclusive to this group (Table S3). Group 3 of SNPs, related to the differentiation of individuals collected in Espírito Santo (*E. edulis_*ALE, *E. edulis_*GUA, *E. edulis_*MI, Santa Marta, *E. edulis_*RN, Tiller, Hybrid_EO, Possible hybrid), presented the highest number of GOs related to the cellular component (8), 10 to the biological process component, and 14 to molecular function. Group 4 of SNPs, differentiating *E. edulis* individuals from AL, presented the lowest number of GOs related to cellular components (3) and the same amount to biological process and molecular function (13). Group 5 of SNPs, differentiating individuals from RJ and RS, presented 12 GOs related to biological processes, one related to the response to abiotic stimulus and response to stress, 7 to cellular components, and 13 to molecular function (Table S3). The nonredundant SNPs selected in each group are shown in Figure S2.

## 4 Discussion

The analyses of genetic diversity and structure and functional annotation of SNPs in different morphotypes of *Euterpe edulis* from Espírito Santo revealed: (1) high genetic differentiation of *E. espiritosantensis* in relation to the individuals from different regions of the state and country; (2) four genetic groups in Espírito Santo; (3) the Santa Marta morphotype was structured; however, it shares with the other populations of Espírito Santo; (4) the individuals that progeny and the possible interspecific hybrids were genetically close; (5) SNPs differentiating the genetic groups were annotated regions of putative candidate genes related to abiotic and biotic stress (Table S3); (6) *Euterpe espiritosantensis* is phylogenetically different from the other populations of *E. edulis* from Espírito Santo; (7) some hybrid individuals share genomic regions with *E. oleracea* , *E. precatoria* , and *E. edulis*.

### 4.1 Genetic Diversity and Structuring of Different Morphotypes

The morphotypes of *E. edulis* from Espírito Santo presented a variable genomic profile of SNPs, with groups of genotypically diverse and little variable SNPs, often almost fixed, depending on the morphotype. These SNPs may indicate a genetic differentiation over time caused by evolutionary events such as genetic drift, gene flow, or founder effect (Wellenreuther et al. 2019). The existence of SNPs with genotypes exclusive to groups of morphotypes or populations may indicate local adaptation to specific environmental conditions (Ren et al. 2013). Here we focused the annotation of a few SNPs only on the coding regions and phylogenetically conserved regions among genera of Arecacea. However, the potential key role of noncoding SNPs in gene expression regulation is also necessary to evaluate. Then an analysis of SNPs in noncoding regions (e.g., promoters, enhancers) would provide a more comprehensive understanding of genetic adaptation.

The tillering morphotype and those reported as interspecific hybrids *E. edulis × E. oleracea* (Hybrid_EO) clustered together and presented a similar genomic profile. According to Bovi et al. (1987) and Carvalho (2016), natural pollination between *E. edulis* and *E. oleracea* can give rise to individuals that present multiple trunks (tillering), hearts of palm with superior size, texture, and flavor and that thrive under high sunlight. Given these characteristics, the Agronomic Institute of Campinas—IAC began to produce interspecific hybrids for commercial production of hearts of palm (Bovi et al. 1987); however, they discontinued the production. It is important to note that hybrid plants produce bunches in the field without fruit, and some individuals also present tillering. However, whether tillering is a common characteristic in the morphotype has not yet been explained.

In the analysis of the genomic profile of the *E. espiritosantensis* morphotype, it was possible to detect a group of SNPs in heterozygosity that differentiates this morphotype from the others. These genetic and genomic differentiations, combined with the morphological differences presented in different studies, support the classification of this morphotype as an ecotype (de Almeida et al. 2024; Wendt et al. 2011). The definition of ecotype established by Hufford and Mazer (2003) states that ecotypes are distinct genotypes (or populations) within a species, resulting from adaptation to local environmental conditions, capable of crossing with other ecotypes of the same species.

The Santa Marta morphotype, which shows morphological variation compared with typical *E. edulis* individuals, also exhibits genetic structuring. Individuals from Santa Marta occur at altitudes between 620 and 870 m, with mean annual temperatures ranging from 11.5°C to 30.3°C (Incaper 2020). This morphological and genetic differentiation may indicate phenotypic plasticity associated with local adaptation to environmental factors. Phenotypic plasticity has also been documented in other studies addressing the distribution of the species in the Atlantic Forest (de Almeida et al. 2024; Brancalion et al. 2018), suggesting that this trait may facilitate the geographic expansion of *E. edulis* into biogeographically similar ecosystems despite edaphic differences.

The low genetic diversity values (*Ho* = 0.067 and *He* = 0.070) found for the *E. espiritosantensis* ecotype indicate allele fixation and homozygosity, which may be due to reduced gene flow and/or geographic isolation. The inbreeding coefficient (*Fis* = 0.100) may indicate that *E. espiritosantensis* may have undergone a recent historical event, which caused a reduction in its effective population size and subsequent expansion from a limited number of individuals (Brancalion et al. 2018; Carvalho et al. 2017). For *E. espiritosantensis*, it is also essential to report the presence of only two genotypes, with the almost complete absence of one of the expected homozygotes per SNP locus, besides a high number of *loci* in homozygosity. This situation differs from similar and isolated populations, such as from Alagoas and DF, which present SNPs with genotypes in homozygosity in general, with a relatively low number of homozygotes. The high homozygosity of *E. espiritosantensis* suggests the occurrence of lineages that underwent recent hybridization, with few *loci* in heterozygosity.

The Tillering (*Fis* = 0.223) and Hybrid_EO (*Fis* = 0.238) morphotypes presented the highest inbreeding coefficient values. Since these morphotypes originated from hybridization, more heterozygous individuals were expected; however, the *Fis* values for both indicate a high rate of homozygous *loci*. The increase in the number of homozygous loci may be related to the inbreeding that the morphotypes may be suffering over time, since they are isolated populations.

The genetic structure of *E. espiritosantensis* morphotype highlights the isolation of the population and the absence of gene flow with other populations in Espírito Santo. Several factors may be influencing the isolation of this morphotype. In the literature, studies report a difference in the flowering peak between *E. edulis* and *E. espiritosantensis*, and distinct patterns of male and female flower production that may limit gene flow (Wendt et al. 2011). In addition, the difference in altitude between the *E. edulis* morphotype and the ecotype may act as a physical barrier, preventing gene flow. However, the low diversity in this morphotype that occurs in a limited region should highlight the region as a priority preservation area of this peculiar genetic pool.

The comparison of the morphotypes of Espírito Santo with the Brazil populations showed that isolation directly influences genetic diversity due to the reduction of gene flow and consequently the increase of inbreeding, as was possible to observe in the population of the Federal District (de Almeida et al. 2024; Carvalho et al. 2015). In contrast, according to the authors, the population of Rio Grande do Sul presented the highest values of *Ho* and *He*, indicating a more significant alleles exchange and genetic variability. The genetic structure analysis between the north/south and northeast/central‐north groups revealed higher differences between the most isolated populations, where gene flow is reduced. This observation reinforces how population isolation due to habitat fragmentation, for example, negatively affects genetic diversity through drift and inbreeding (de Almeida et al. 2024; Carvalho et al. 2021).

The high genetic differentiation of *E. espiritosantensis* as an exclusive gene cluster, compared with samples from Espírito Santo and the country, was evident in this study, with low genetic diversity and practically no homozygous genotypes. For heterozygous SNPs, these individuals are a source of new alleles for the other populations studied. This morphotype occurs only in the municipality of Santa Teresa, in the form of patches in medium and low forests on the top of slopes, upper edge of escarpments, and bottom of valleys (Fernandes 1989). The altitudes varied between 700 and 1000 m, mainly where quartzite‐sandy soil occurs (Fernandes 1989). The natural population isolation due to factors such as altitude can reduce diversity by reducing gene flow and increase inbreeding levels through mating between related individuals (de Almeida et al. 2024; Carvalho et al. 2015). Another critical aspect of the *E. espiritosantensis* is its restricted occurrence in a specific region of the state (Fernandes 1989; Wendt et al. 2011). Although the collection site is a preservation area, the low genetic diversity demonstrates the importance of conserving this differential gene pool for the species.

The integration of genetic structure with geographic information using a spatially explicit approach corroborates the patterns inferred by nonspatial analyses and provides additional support for the biological interpretation of the identified clusters. The strong spatial confinement and genetic differentiation of *E. espiritosantensis* are consistent with its restricted distribution, ecological specificity, and reduced gene flow, reinforcing its classification as an ecotype within *E. edulis* . In contrast, the extensive spatial overlap and admixture observed among typical *E. edulis* populations suggest ongoing or historical connectivity across municipalities in Espírito Santo. The spatial association between tillering individuals and Hybrid_EO further supports the hypothesis of a shared genetic origin linked to hybridization events. Although higher *K* values reveal finer‐scale structure, the lack of clear geographic segregation at *K* = 5 and 6 indicates that *K* = 4 represents the most parsimonious model, capturing biologically meaningful patterns without over‐partitioning the genetic variation. A study carried out by Coelho et al. (2020) reported a low number of homozygotes (*Ho* = 0.51–0.55) and an excess of heterozygotes (*He* = 0.67–0.75) for three populations of red palm heart, named by the authors as *Euterpe espiritosantensis*. However, the populations used by the authors were collected in southern Bahia, where no records of this morphotype exist. Besides, there is a mistake in the description of the material, as the popular name “red palm heart” refers to the color of the inflorescence and not to the palm heart that has a yellow coloration, as described by Fernandes (1989) in the article that described *Euterpe espiritosantensis*.

These data are unprecedented since previous studies carried out with SSR, although they detected low to intermediate genetic differentiation between populations in the state (Carvalho et al. 2020; Mengarda et al. 2022; Pereira et al. 2022); the *Fst* values for *E. espiritosantensis* detected in this study (between 0.56 and 0.62) are much higher than those previously reported, even considering the differences between markers (de Almeida et al. 2024). It is also crucial to highlight the SNP markers' relevance in differentiating among the groups and SSR differentiating among the individuals (de Almeida et al. 2024). According to Garbin et al. (2017), Espírito Santo has six phytoecological regions in a small territorial extension. Variations in altitude and vegetation types can directly influence genetic diversity since ecological barriers can restrict the dispersal of pollen or seeds, causing populations to differentiate over time. Furthermore, different selective pressures can lead to the random fixation of different allelic sets in populations through genetic drift.

### 4.2 Phylogenetic Analysis

*Euterpe espiritosantensis* presents phylogenetic evidence of a recent evolutionary process that differentiated this group from the other populations of *Euterpe edulis* evaluated in Brazil and Espírito Santo. The fact that this population was initially described as a distinct species and later synonymized with *E. edulis* and presents ecological, morphological, genetic, and phenological differences that prevent and/or reduce gene flow with the other populations corroborates the hypothesis of sympatric speciation proposed by Wendt et al. (2011).

Pereira et al. (2022) described the populations of Brasília, Alagoas, and Bahia as well‐structured, indicating geographic isolation. de Almeida et al. (2024) used 2227 neutral loci and reported low values of expected heterozygosity and observed heterozygosity, besides a high inbreeding, indicating low gene flow among these populations. Phylogenetic data reinforce this hypothesis of population isolation since populations were in different clades with high support values.

Several factors lead to genetic changes in *Euterpe edulis* populations. Defaunation is crucial due to the decrease in long‐distance seed dispersal (Carvalho et al. 2016). Geographic isolation contributes to reducing gene flow between populations, increasing mating between related individuals and, consequently, levels of inbreeding (Carvalho et al. 2017; Gaiotto 2003; Pérez‐Alquicira et al. 2023). These factors, associated with habitat fragmentation and a decrease in dispersers and pollinators, lead to long‐term differentiation of populations and even speciation (Gaiotto 2003).

The phylogenetic tree showed that profiling individuals (Tiller) shared genomic regions with *E. oleracea* and *E. precatoria*, since some individuals were grouped with an outgroup, corroborating the hybridization hypothesis. Hybridization may explain the tillering behavior in one of the populations analyzed, their low production and small fruits.

The grouping of the hybrid of Vargem Alta (Hybrid_EO) with *E. oleracea* and *E. precatoria* confirms the origin of that population that was developed from interspecific cross‐breeding for the production of palm hearts, as reported by the producer and found in the study carried out by Bovi et al. (1987). However, both morphotypes are also clustered with *E. edulis* , indicating that there may be genomic region exchanges between the morphotypes and the species.

### 4.3 SNPs Functional Annotation

The functional annotation of the SNP groups that differentiated groups of individuals revealed that GOs related to stress response, external stimuli, and anatomical structural development are candidates to be involved in the phenotypic differentiation between morphotypes. The characterization of GOs shows a predominance of biological processes, followed by molecular function in the four groups. The GO terms related to metabolic, cellular, and biological processes were highly enriched.

The GOs identified in group 1 of SNPs that differentiate *E. edulis* individuals collected in DF are related to the response to endogenous and chemical stimuli. Serine/threonine‐protein phosphatase belongs to the PPP family. It is essential in different pathways of plant metabolism and development, such as the development of meristematic tissues and intracellular regulation of response to brassinosteroids (Uhrig et al. 2013).

Group 2 of SNPs, composed of SNPs differentiating *Euterpe espiritosantensis*, showed GOs related to light stimuli, anatomical structure development, postembryonic development, reproduction, and stress response. The GIGANTEA (GI) gene was annotated, which is involved in regulating the circadian rhythm and controlling photoperiodic flowering (https://www.ebi.ac.uk/interpro/). Photoperiodic control of flowering is a vital developmental process in plants because it is directly related to successful reproduction (Park et al. 2016). In *Arabidopsis*, mutations in the GIGANTEA gene delay flowering in long days, but the effects are minimal in short days (Fujiwara et al. 2008). The SNP related to this gene that differs from *E. espiritosantensis* corroborates the work of Wendt et al. (2011). These authors pointed out the overlap between the flowering period of *E. espiritosantensis* and *E. edulis* , but with distinctly different flowering peaks. Analyses also identified the DNA excision repair protein ERCC‐1 in this region. ERCC‐1 performs excision repair of damaged nucleotides and can acquire relevance under oxidative stress conditions (https://www.ebi.ac.uk/interpro/). In wheat, it is involved in the repair of damage induced by ultraviolet light and cold acclimation (Jaikumar et al. 2020).

Group 5, which includes individuals from RJ and RS, presented GOs related to the response to abiotic stimuli. The DnaJ gene family responds to cellular stress, especially hyperosmotic and heat shock conditions. During stress, genes in this family act as chaperones to repair polypeptide unfolding and protein aggregation. In addition, this family is also related to the targeting of selected proteins for degradation (https://www.ebi.ac.uk/interpro/). In *Arabidopsis*, proteins in this family are involved in homeostasis, regulating folding and unfolding, assembly, and translocation under stress conditions (Jia et al. 2021).

Identifying genes and proteins in a species that may be involved in morphological differentiation, as performed in the present study, is essential for understanding its environmental interaction, aiming at improving productivity and resistance to adverse environmental conditions. This work provides important results on SNPs that differentiate *E. edulis* morphotypes related to development and responsiveness to external stimuli that can aid in the management of the species in different locations.

## 5 Conclusions

Analyses of genetic diversity and structuring showed differences between the *Euterpe* morphotypes found in Espírito Santo, including populations such as *Euterpe espiritosantensis* presenting high levels of differentiation.

This study revealed insights into the genetic diversity and structuring of the different morphotypes of *Euterpe edulis* in Espírito Santo, contributing to the understanding of the genetic and ecological complexity of the species. The analyses demonstrated high genetic differentiation of *Euterpe espiritosantensis* to populations from other regions of the state and Brazil, confirming its status as a phylogenetically distinct group. The region displayed four genetic groups, highlighting the intrinsic local diversity. The topology of the phylogenetic tree supports the hypothesis of hybrid individuals between *E. edulis* and *E. oleracea* and *E. precatoria* , which may suggest the occurrence of genetic introgression events. However, targeted analyses would be necessary to confirm this hypothesis. The genetic relationship between profiling individuals and possible interspecific hybrids reinforces the role of hybridization in the genetic diversification of the species. Differentiating SNPs between the genetic groups identified genes associated with abiotic and biotic stress, evidencing potential local adaptations. These results reinforce the importance of preserving *E. edulis* and its morphotypes, especially under environmental threats, and suggest the need for specific management strategies that consider populations' genetic variability and evolutionary dynamics.

## Author Contributions

**Jônatas Gomes Santos:** conceptualization (equal), formal analysis (equal), methodology (equal), resources (equal), writing – original draft (equal), writing – review and editing (equal). **Francine Alves Nogueira de Almeida:** methodology (equal), writing – review and editing (equal). **Hélio de Queiroz Boudet‐Fernandes:** investigation (supporting), resources (supporting), validation (supporting). **Pedro Henrique Dias dos Santos:** methodology (equal). **Suelane Costa dos Santos:** methodology (equal), software (equal), writing – review and editing (equal). **Miquéias Fernandes:** methodology (equal). **Adésio Ferreira:** funding acquisition (equal), methodology (equal), project administration (equal), supervision (equal). **Marcia Flores da Silva Ferreira:** conceptualization (equal), funding acquisition (equal), methodology (equal), project administration (equal), resources (equal), supervision (equal), writing – review and editing (equal).

## Funding

This work was supported by Fundação de Amparo à Pesquisa e Inovação do Espírito Santo. Coordenação de Aperfeiçoamento de Pessoal de Nível Superior; Conselho Nacional de Desenvolvimento Científico e Tecnológico.

## Conflicts of Interest

The authors declare no conflicts of interest.

## Supporting information


**Figure S1:** ece372921‐sup‐0001‐Supinfo.zip.

**Data S1:** ece372921‐sup‐0002‐DataS1.xlsx.

## Data Availability

All the required data are uploaded as Supporting Information.

## References

[ece372921-bib-0001] Afgan, E. , A. Nekrutenko , B. Grüning , et al. 2022. “The Galaxy Platform for Accessible, Reproducible and Collaborative Biomedical Analyses: 2022 Update.” Nucleic Acids Research 50, no. W1: W345–W351. 35446428 10.1093/nar/gkac247 PMC9252830

[ece372921-bib-0002] Aleksander, S. A. , J. Balhoff , S. Carbon , et al. 2023. “The Gene Ontology Knowledgebase in 2023.” Genetics 224, no. 1: iyad031. 36866529 10.1093/genetics/iyad031 PMC10158837

[ece372921-bib-0055] Barroso, R. M. , A. Reis , and N. Hanazaki . 2010. “Ethnoecology and Ethnobotany of the Juçara Palm (*Euterpe edulis* Martius) in Quilombola Communities of the Ribeira River Valley, São Paulo.” Acta Botânica Brasílica 24, no. 2: 518–528.

[ece372921-bib-0003] Bovi, M. L. A. , G. G. Junior , and L. A. Sáes . 1987. “Híbridos interespecíficos de palmiteiro (*Euterpe Oleracea* X *Euterpe Edulis*).” Bragantia 2: 343–363.

[ece372921-bib-0004] Brancalion, P. H. S. , G. C. X. Oliveira , M. I. Zucchi , et al. 2018. “Phenotypic Plasticity and Local Adaptation Favor Range Expansion of a Neotropical Palm.” Ecology and Evolution 8, no. 15: 7462–7475. 30151163 10.1002/ece3.4248 PMC6106193

[ece372921-bib-0005] Carvalho, C. S. , L. Ballesteros‐Mejia , M. C. Ribeiro , et al. 2017. “Climatic Stability and Contemporary Human Impacts Affect the Genetic Diversity and Conservation Status of a Tropical Palm in the Atlantic Forest of Brazil.” Conservation Genetics 18, no. 2: 467–478.

[ece372921-bib-0059] Carvalho, C. S. , M. Galetti , R. G. Colevatti , and P. Jordano . 2016. “Defaunation Leads to Microevolutionary Changes in a Tropical Palm.” Scientific Reports 6, no. 1: 31957. 10.1038/srep31957. 27535709 PMC4989191

[ece372921-bib-0006] Carvalho, C. S. , C. García , M. S. Lucas , P. Jordano , and M. Corrêa Côrtes . 2021. “Extant Fruit‐Eating Birds Promote Genetically Diverse Seed Rain, but Disperse to Fewer Sites in Defaunated Tropical Forests.” Journal of Ecology 109, no. 2: 1055–1067.

[ece372921-bib-0007] Carvalho, C. S. , M. C. Ribeiro , M. C. Côrtes , M. Galetti , and R. G. Collevatti . 2015. “Contemporary and Historic Factors Influence Differently Genetic Differentiation and Diversity in a Tropical Palm.” Heredity 115, no. 3: 216–224. 25873150 10.1038/hdy.2015.30 PMC4814231

[ece372921-bib-0008] Carvalho, M. S. , M. Ferreira , W. B. S. Oliveira , et al. 2020. “Genetic Diversity and Population Structure of *Euterpe eulis* by REML/BLUP Analysis of Fruit Morphology and Microsatellite Markers Genetic Diversity and Population Structure of *Euterpe edulis* by REML/BLUP Analysis of Fruit Morphology and Microsatellite Markers.” Crop Breeding and Applied Biotechnology 20, no. 4: 31662048.

[ece372921-bib-0009] Caye, K. , T. M. Deist , H. Martins , O. Michel , and O. François . 2016. “TESS3: Fast Inference of Spatial Population Structure and Genome Scans for Selection.” Molecular Ecology Resources 16, no. 2: 540–548. 10.1111/1755-0998.12471. 26417651

[ece372921-bib-0010] Cerqueira, A. F. , A. S. Santos , O. de Alencar , et al. 2022. “Landscape Conservation and Maternal Environment Affect Genetic Diversity and the Physiological Responses of *Euterpe edulis* (Arecaceae) Progenies to Light Availability.” Environmental and Experimental Botany 194: 104722.

[ece372921-bib-0011] Cock, P. J. A. , T. Antao , J. T. Chang , et al. 2009. “Biopython: Freely Available Python Tools for Computational Molecular Biology and Bioinformatics.” Bioinformatics 25, no. 11: 1422–1423. 19304878 10.1093/bioinformatics/btp163 PMC2682512

[ece372921-bib-0012] Coelho, G. M. , A. S. Santos , I. P. P. de Menezes , et al. 2020. “Genetic Structure Among Morphotypes of the Endangered Brazilian Palm *Euterpe edulis* Mart (Arecaceae).” Ecology and Evolution 10, no. 12: 6039–6048. 32607211 10.1002/ece3.6348 PMC7319139

[ece372921-bib-0057] de Almeida, F. A. N. , J. G. Santos , A. G. Pereira , et al. 2024. “Genetic Diversity Analysis of *Euterpe edulis* Based on Different Molecular Markers.” Tree Genetics & Genomes 20, no. 5. 10.1007/s11295-024-01663-9.

[ece372921-bib-0013] De Carvalho, L. M. J. , A. A. Esmerino , and J. L. V. De Carvalho . 2022. “Jussaí (*Euterpe edulis*): A Review.” Food Science and Technology 42: e08422. 10.1590/fst.08422.

[ece372921-bib-0056] de Medeiros Cardozo, L. , F. A. N. de Almeida , V. S. Fioresi , et al. 2025. “Conserved Stress‐Responsive Genes Involved in the Early Development of *Euterpe edulis* .” Scientific Reports 15, no. 1: 22862. 10.1038/s41598-025-01436-x. 40592938 PMC12214915

[ece372921-bib-0014] De Moraes, M. C. , L. H. G. Mengarda , G. B. Canal , et al. 2020. “Genetic Diversity in Matrices and Progenies of *Euterpe Edulis* Mart. In Managed Area and in Natural Populations by Microsatellites Markers.” Ciência Florestal 30, no. 2: 583–594.

[ece372921-bib-0015] Ding, Z. , L. Fu , B. Wang , et al. 2023. “Metabolic GWAS‐Based Dissection of Genetic Basis Underlying Nutrient Quality Variation and Domestication of Cassava Storage Root.” Genome Biology 24, no. 1: 289. 38098107 10.1186/s13059-023-03137-y PMC10722858

[ece372921-bib-0054] Doyle, J. J. , and J. L. Doyle . 1990. “Isolation of Plant DNA From Fresh Tissue.” Focus 12: 13–15.

[ece372921-bib-0016] Dwiningsih, Y. , M. Rahmaningsih , and J. Alkahtani . 2020. “Development of Single Nucleotide Polymorphism (SNP) Markers in Tropical Crops.” Advance Sustainable Science, Engineering and Technology 2, no. 2: 1–14. 10.26877/asset.v2i2.6279.

[ece372921-bib-0017] El‐Gebali, S. , J. Mistry , A. Bateman , et al. 2019. “The Pfam Protein Families Database in 2019.” Nucleic Acids Research 47, no. D1: D427–D432. 30357350 10.1093/nar/gky995 PMC6324024

[ece372921-bib-0018] Fernandes, H. Q. B. 1989. “Uma nova espécie de Euterpe (Palmae ‐ Arecoideae ‐ Areceae) do Brasil.” Acta Botânica Brasílica 3, no. 2: 43–49.

[ece372921-bib-0019] Ferreira, M. E. , and D. Grattapaglia . 1998. “Introdução ao uso de marcadores moleculares em análise genética.”

[ece372921-bib-0020] Frichot, E. , and O. François . 2015. “LEA: An R Package for Landscape and Ecological Association Studies.” Methods in Ecology and Evolution 6, no. 8: 925–929.

[ece372921-bib-0021] Fujiwara, S. , A. Oda , R. Yoshida , et al. 2008. “Circadian Clock Proteins LHY and CCA1 Regulate SVP Protein Accumulation to Control Flowering in *Arabidopsis* .” Plant Cell 20, no. 11: 2960–2971. 19011118 10.1105/tpc.108.061531 PMC2613671

[ece372921-bib-0022] Gaiotto, F. A. 2003. “Genetic Structure, Mating System, and Long‐Distance Gene Flow in Heart of Palm (*Euterpe edulis* Mart.).” Journal of Heredity 94, no. 5: 399–406. 14557393 10.1093/jhered/esg087

[ece372921-bib-0061] Galetti, M. , V. B. Zipparro , and L. P. C. Morellato . 1999. “Fruit Phenology and Frugivory on the Palm *Euterpe edulis* in a Lowland Atlantic Forest of Brazil.” Ecotropica 5: 115–122.

[ece372921-bib-0023] Garbin, M. L. , F. Z. Saiter , T. T. Carrijo , et al. 2017. “Breve histórico e classificação da vegetação capixaba.” Rodriguésia 68, no. 5: 1883–1894.

[ece372921-bib-0062] Genini, J. , M. Galetti , and L. P. C. Morellato . 2009. “Fruiting Phenology of Palms and Trees in an Atlantic Rainforest Land‐Bridge Island.” Flora – Morphology, Distribution, Functional Ecology of Plants 204, no. 2: 131–145. 10.1016/j.flora.2008.01.002.

[ece372921-bib-0024] Goudet, J. 2005. “hierfstat, a Package for R to Compute and Test Hierarchical F‐Statistics.” Molecular Ecology Notes 5, no. 1: 184–186.

[ece372921-bib-0025] Gruber, B. , P. J. Unmack , O. F. Berry , and A. Georges . 2018. “dartr: An R Package to Facilitate Analysis of SNP Data Generated From Reduced Representation Genome Sequencing.” Molecular Ecology Resources 18, no. 3: 691–699. 29266847 10.1111/1755-0998.12745

[ece372921-bib-0058] Hufford, K. M. , and S. J. Mazer . 2003. “Plant Ecotypes: Genetic Differentiation in the Age of Ecological Restoration.” Trends in Ecology & Evolution 18, no. 3: 147–155. 10.1016/s0169-5347(03)00002-8.

[ece372921-bib-0060] Instituto Capixaba de Pesquisa, Assistência Técnica e Extensão Rural (INCAPER) . 2020. “Programa Estadual de Assistência Técnica e Extensão Rural – PROATER 2020.” Vitória, ES: Autor.

[ece372921-bib-0026] Jaikumar, N. S. , K. M. Dorn , D. Baas , B. Wilke , C. Kapp , and S. S. Snapp . 2020. “Nucleic Acid Damage and DNA Repair Are Affected by Freezing Stress in Annual Wheat (*Triticum aestivum*) and by Plant Age and Freezing in Its Perennial Relative (*Thinopyrum intermedium*).” American Journal of Botany 107, no. 12: 1693–1709. 33340368 10.1002/ajb2.1584

[ece372921-bib-0027] Jia, T. , F. Li , S. Liu , J. Dou , and T. Huang . 2021. “DnaJ Proteins Regulate WUS Expression in Shoot Apical Meristem of Arabidopsis.” Plants 10, no. 1: 136. 33445404 10.3390/plants10010136 PMC7827474

[ece372921-bib-0028] Jones, P. , D. Binns , H. Y. Chang , et al. 2014. “InterProScan 5: Genome‐Scale Protein Function Classification.” Bioinformatics 30, no. 9: 1236–1240. 24451626 10.1093/bioinformatics/btu031 PMC3998142

[ece372921-bib-0029] Kamvar, Z. N. , J. F. Tabima , and N. J. Grünwald . 2014. “Poppr: An R Package for Genetic Analysis of Populations With Clonal, Partially Clonal, and/or Sexual Reproduction.” PeerJ 2: e281. 24688859 10.7717/peerj.281 PMC3961149

[ece372921-bib-0030] Keenan, K. , P. McGinnity , T. F. Cross , W. W. Crozier , and P. A. Prodöhl . 2013. “diveRsity: An R Package for the Estimation and Exploration of Population Genetics Parameters and Their Associated Errors.” Methods in Ecology and Evolution 4, no. 8: 782–788.

[ece372921-bib-0031] Mantovani, A. , and P. Morellato . 2000. “Fenologia da floração, frutificação, mudança foliar e aspectos da biologia floral.” 23–38.

[ece372921-bib-0032] McCarthy, F. M. , S. M. Bridges , N. Wang , et al. 2007. “AgBase: A Unified Resource for Functional Analysis in Agriculture.” Nucleic Acids Research 35: D599–D603. 17135208 10.1093/nar/gkl936 PMC1751552

[ece372921-bib-0033] Mengarda, L. H. G. , G. B. Canal , A. Ferreira , et al. 2022. “Genetic Diversity of juçara Palm: An Alternative for Selection and Conservation in Cash Crop for Fruit Production.” Frontiers in Forests and Global Change 5: 859081. 10.3389/ffgc.2022.859081.

[ece372921-bib-0034] Nei, M. , and W. H. Li . 1979. “Mathematical Model for Studying Genetic Variation in Terms of Restriction Endonucleases.” Proceedings of the National Academy of Sciences 76, no. 10: 5269–5273. 10.1073/pnas.76.10.5269 PMC413122 291943

[ece372921-bib-0035] O'Leary, N. A. , M. W. Wright , J. R. Brister , et al. 2016. “Reference Sequence (RefSeq) Database at NCBI: Current Status, Taxonomic Expansion, and Functional Annotation.” Nucleic Acids Research 44, no. D1: D733–D745. 26553804 10.1093/nar/gkv1189 PMC4702849

[ece372921-bib-0036] Park, H. J. , W. Y. Kim , J. M. Pardo , and D. J. Yun . 2016. “Molecular Interactions Between Flowering Time and Abiotic Stress Pathways.” International Review of Cell and Molecular Biology 327: 371–412. 27692179 10.1016/bs.ircmb.2016.07.001

[ece372921-bib-0037] Patterson, N. , A. L. Price , and D. Reich . 2006. “Population Structure and Eigen Analysis.” PLoS Genetics 2, no. 12: e190. 17194218 10.1371/journal.pgen.0020190 PMC1713260

[ece372921-bib-0038] Pereira, A. G. , M. F. da Silva Ferreira , T. C. da Silveira , et al. 2022. “Patterns of Genetic Diversity and Structure of a Threatened Palm Species (*Euterpe edulis* Arecaceae) From the Brazilian Atlantic Forest.” Heredity 129, no. 3: 161–168. 35697755 10.1038/s41437-022-00549-7 PMC9411632

[ece372921-bib-0039] Pérez‐Alquicira, J. , E. V. Wehncke , G. A. García‐Loza , et al. 2023. “Geographic Isolation and Long‐Distance Gene Flow Influence the Genetic Structure of the Blue Fan Palm Brahea Armata (Arecaceae).” Journal of Plant Research 136, no. 3: 277–290. 36905462 10.1007/s10265-023-01445-9

[ece372921-bib-0040] Pritchard, J. K. , M. Stephens , and P. Donnelly . 2000. “Inference of Population Structure Using Multilocus Genotype Data.” Genetics 155, no. 2: 945–959. 10835412 10.1093/genetics/155.2.945 PMC1461096

[ece372921-bib-0053] R Core Team . 2021. “R: A Language and Environment for Statistical Computing.” R Foundation for Statistical Computing. https://www.R‐project.org/.

[ece372921-bib-0063] Ren, J. , L. Chen , D. Sun , et al. 2013. “SNP‐Revealed Genetic Diversity in Wild Emmer Wheat Correlates With Ecological Factors.” BMC Evolutionary Biology 13, no. 1: 169. 10.1186/1471-2148-13-169. 23937410 PMC3751623

[ece372921-bib-0042] Sansaloni, C. , C. Petroli , D. Jaccoud , et al. 2011. “Diversity Arrays Technology (DArT) and Next‐Generation Sequencing Combined: Genome‐Wide, High Throughput, Highly Informative Genotyping for Molecular Breeding of Eucalyptus.” BMC Proceedings 5, no. S7: P54.22373051

[ece372921-bib-0043] Thomas, P. D. , D. Ebert , A. Muruganujan , T. Mushayahama , L. P. Albou , and H. Mi . 2022. “PANTHER: Making Genome‐Scale Phylogenetics Accessible to All.” Protein Science 31, no. 1: 8–22. 34717010 10.1002/pro.4218 PMC8740835

[ece372921-bib-0044] Uhrig, R. G. , A. M. Labandera , and G. B. Moorhead . 2013. “Arabidopsis PPP Family of Serine/Threonine Protein Phosphatases: Many Targets but Few Engines.” Trends in Plant Science 18: 505–513. 23790269 10.1016/j.tplants.2013.05.004

[ece372921-bib-0051] Vianna, S. A. 2020. “Euterpe in Flora do Brasil 2020.” Jardim Botânico do Rio de Janeiro. https://floradobrasil2020.jbrj.gov.br/FB15711.

[ece372921-bib-0052] Weir, B. S. , and C. C. Cockerham . 1984. “Estimating F‐Statistics for the Analysis of Population Structure.” Evolution 38, no. 6: 1358–1370. 10.1111/j.1558-5646.1984.tb05657.x. 28563791

[ece372921-bib-0045] Wellenreuther, M. , C. Mérot , E. Berdan , and L. Bernatchez . 2019. “Going Beyond SNPs: The Role of Structural Genomic Variants in Adaptive Evolution and Species Diversification.” Molecular Ecology 28, no. 6: 1203–1209. 30834648 10.1111/mec.15066

[ece372921-bib-0046] Wendt, T. , D. D. da Cruz , V. G. Demuner , F. A. G. Guilherme , and H. Boudet‐Fernandes . 2011. “An Evaluation of the Species Boundaries of Two Putative Taxonomic Entities of *Euterpe* (Arecaceae) Based on Reproductive and Morphological Features.” Flora: Morphology, Distribution, Functional Ecology of Plants 206, no. 2: 144–150.

[ece372921-bib-0047] Young, A. , T. Boyle , and T. Brown . 1996. “The Population Genetic Consequences of Habitat Fragmentation for Plants.” Trends in Ecology & Evolution 11, no. 10: 413–418. 21237900 10.1016/0169-5347(96)10045-8

[ece372921-bib-0048] Zhang, Q. , and Z. D. Zhang . 2022. “Protocol for Gene Annotation, Prediction, and Validation of Genomic Gene Expansion.” STAR Protocols 3, no. 4: 101692. 36125934 10.1016/j.xpro.2022.101692 PMC9494284

