# Supplementary material for: Genomic Differentiation, Diversity, and Genetic Structuring of Euterpe edulis Mart. Morphotype in Espírito Santo, Brazil
Source: Ecol Evol. 2026 Jan 20;16(1):e72921. doi: 10.1002/ece3.72921 (PMC12819052; doi:10.1002/ece3.72921)
Supplement: Supplementary file 1 — Figure S1: ece372921‐sup‐0001‐Supinfo.zip. [file ECE3-16-e72921-s002.zip › ece372921-sup-0001-FigureS1-S2-TableS1-S3@18122025_Supplementary material.docx]

Supplementary material

**Figure S1.** Description of the morphotypes of *Euterpe edulis* found in Espírito Santo. The images were obtained during field expeditions. The table displays the identification acronyms used in the text, the main characteristics of each morphotype, and the references that cited these morphotypes.


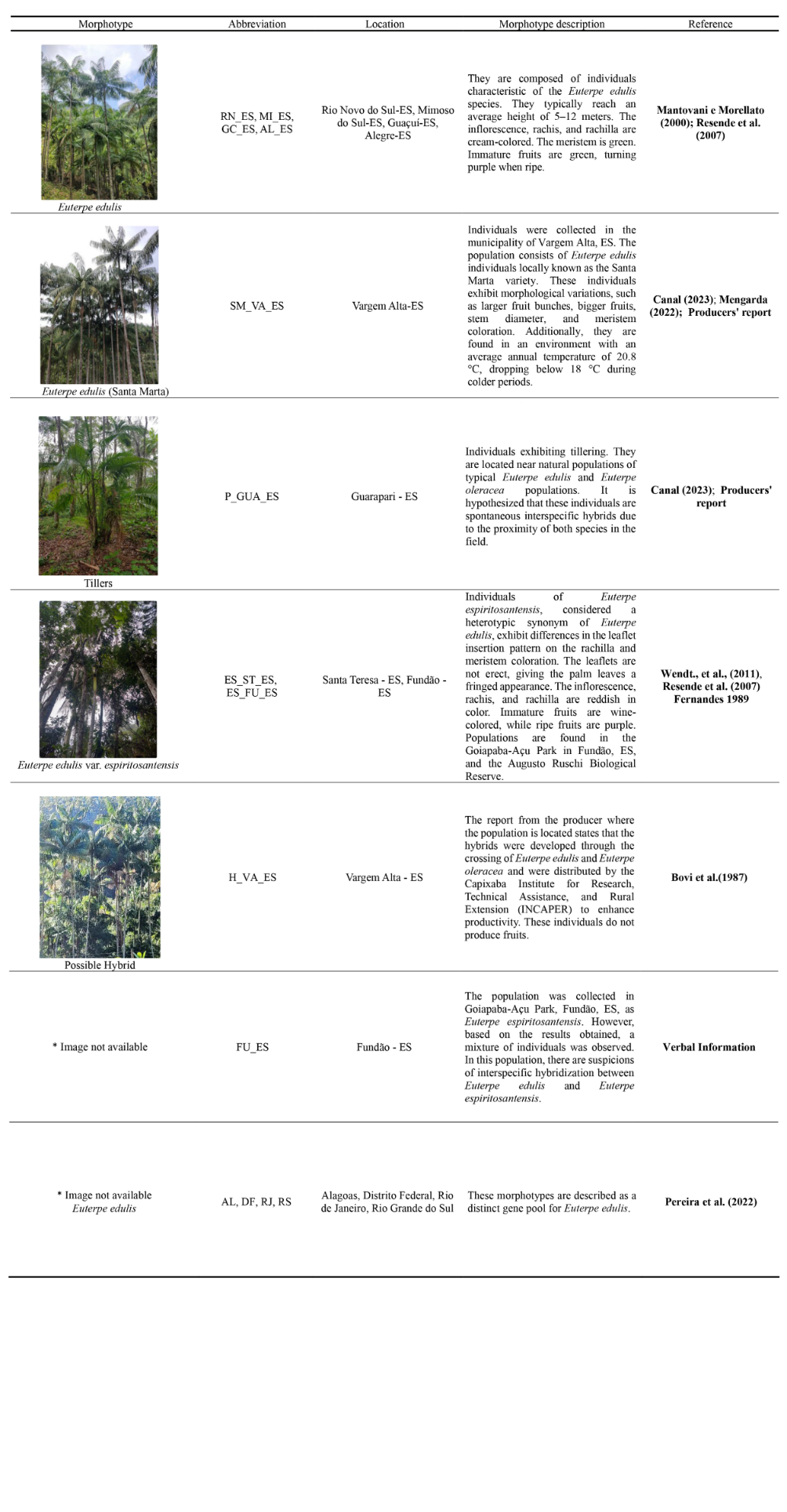


**Figure S2.** Genomic profile of genetic groups of *E. edulis* from Espírito Santo and Brazil, obtained with 154 differentiating SNPs (5 groups of SNPs), with functional annotation. Interestingly, the differentiating SNPs with functional annotation are mostly heterozygous.
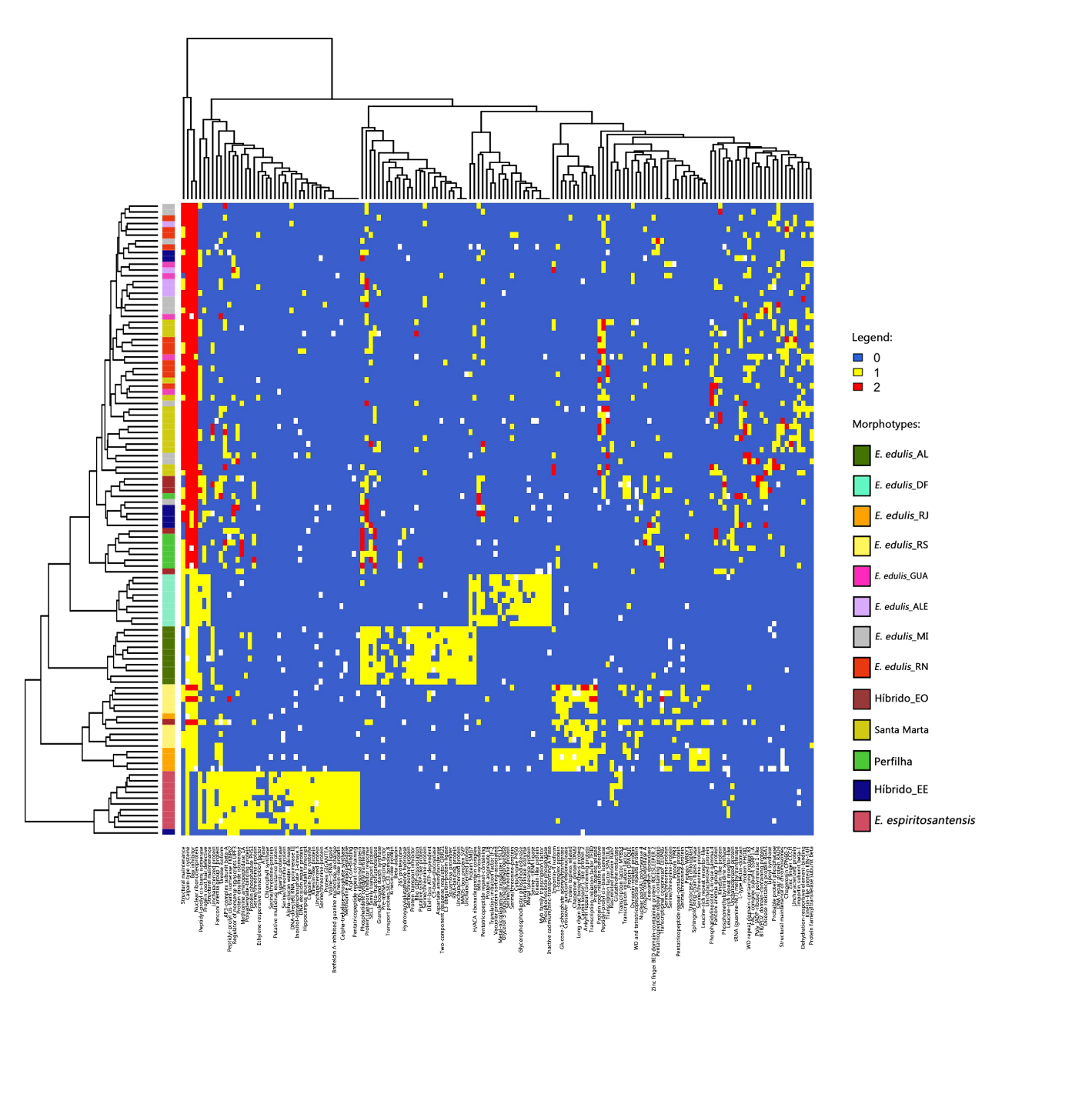


**Figura S3.** Spatially explicit ancestry analysis of *Euterpe edulis* morphotype populations from Espírito Santo inferred with the TESS3 package. (A–C) Individual ancestry coefficients for $K=4$, $K=5$ and $K=6$, respectively, showing bar plots of admixture proportions (left panels) and spatial interpolation of ancestry coefficients across the sampled area (right panels). Each color represents a distinct ancestral genetic cluster, and each vertical bar corresponds to one individual. (D) Geographic localization of sampling sites in Espírito Santo (left) and cross‐validation scores as a function of the number of ancestral populations (right), indicating improved model fit with increasing $K$ values within the tested range.

**
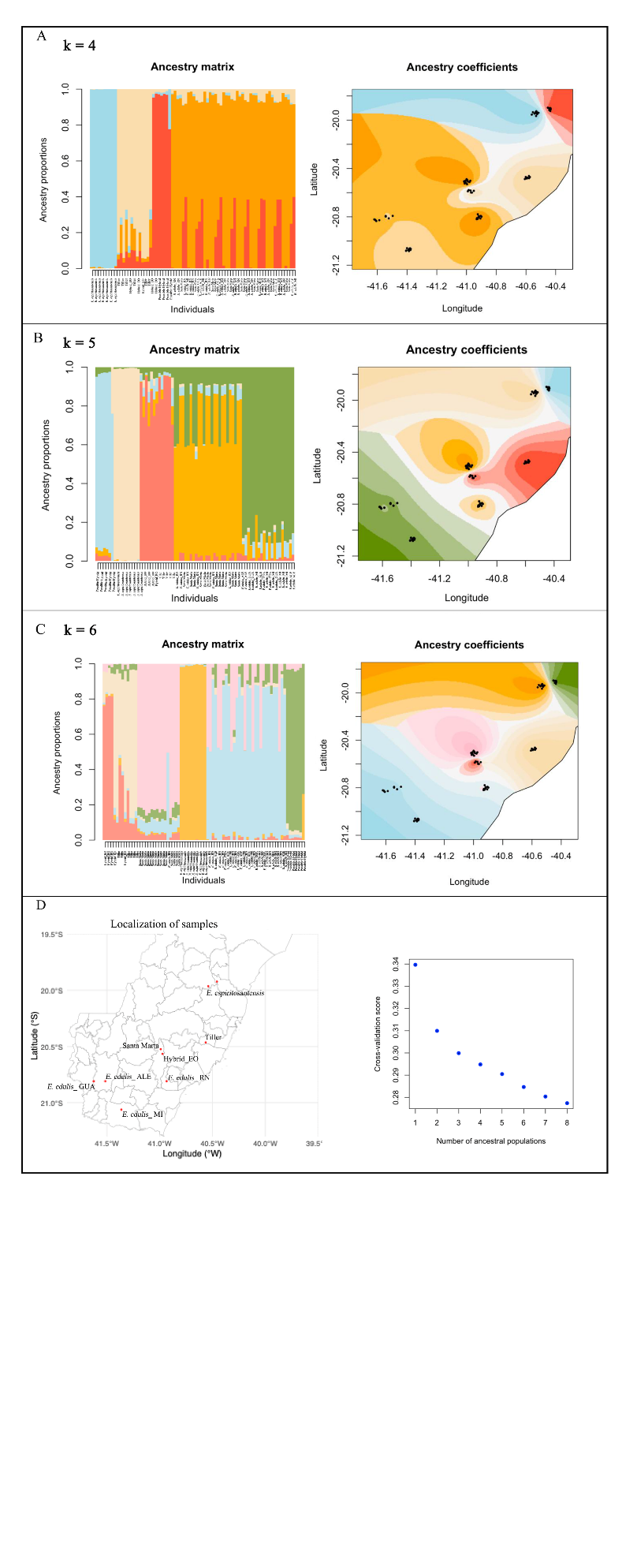
**

| **Number of SNPs** | **Source of Variation** | **Degrees of Freedom** | **Sum of Squares** | **Mean Squares** | **Sigma** | **% Variation** | **Phi** |
| --- | --- | --- | --- | --- | --- | --- | --- |
| 2,984 SNP | Among populations | 12 | 33866.66 | 2822.22 | 320.53 | 66.71 |  |
|  | Within populations | 96 | 15355.52 | 159.95 | 159.95 | 33.29 |  |
|  | Total | 108 | 49222.18 | 455.76 | 480.48 | 100.00 | 0.67 |
| **Number of SNPs** | **Source of Variation** | **Degrees of Freedom** | **Sum of Squares** | **Mean Squares** | **Sigma** | **% Variation** | **Phi** |
| 2,984  SNP | Among morphotypes | 8 | 14149.85 | 1768.73 | 123.95 | 26.13 |  |
|  | Within the morphotypes | 100 | 35037.60 | 350.37 | 350.37 | 73.87 |  |
|  | Total | 108 | 49187.45 | 455.44 | 474.33 | 100.00 | 0.26 |

**Table S1** . Analysis of Molecular Variance (AMOVA) for 2,982 SNPs with different hierarchy levels. The first hierarchical level considered was the populations, allowing a comprehensive study to investigate variations among populations and within the same population. The second analysis level focused on morphotypes, enabling an investigation of variations among morphotypes and within the same morphotype.

**Table S2. Genetic differentiation values (FST) between** *Euterpe edulis* morphotypes in Espírito Santo based on 5,319 SNPs.

|  | AL_ES | FU_ES | ES_ST_ES | GC_ES | H_VA_ES | MI_ES | P_GUA_ES | RN_ES | SM_VA_ES |
| --- | --- | --- | --- | --- | --- | --- | --- | --- | --- |
| AL_ES | 0 | 0.141727 | 0.626406 | 0.002696 | 0.106695 | 0.012512 | 0.180825 | 0.051425 | 0.061809 |
| FU_ES | 0.141727 | 0 | 0.565027 | 0.143861 | 0.123502 | 0.117653 | 0.178436 | 0.167259 | 0.17661 |
| ES_ST_ES | 0.626406 | 0.565027 | 0 | 0.622892 | 0.56903 | 0.598894 | 0.609979 | 0.594804 | 0.585622 |
| GC_ES | 0.002696 | 0.143861 | 0.622892 | 0 | 0.10972 | 0.012553 | 0.182452 | 0.035401 | 0.05237 |
| H_VA_ES | 0.106695 | 0.123502 | 0.56903 | 0.10972 | 0 | 0.113407 | 0.073907 | 0.149794 | 0.16185 |
| MI_ES | 0.012512 | 0.117653 | 0.598894 | 0.012553 | 0.113407 | 0 | 0.174039 | 0.057923 | 0.073837 |
| P_GUA_ES | 0.180825 | 0.178436 | 0.609979 | 0.182452 | 0.073907 | 0.174039 | 0 | 0.20832 | 0.220553 |
| RN_ES | 0.051425 | 0.167259 | 0.594804 | 0.035401 | 0.149794 | 0.057923 | 0.20832 | 0 | 0.041715 |
| SM_VA_ES | 0.061809 | 0.17661 | 0.585622 | 0.05237 | 0.16185 | 0.073837 | 0.220553 | 0.041715 | 0 |

**Table S3** – Non-redundant SNPs annotated for the regions selected in the genomic profile. The table contains the region, SNP identification, gene, mRNA, protein, gene ontology (GO), annotation, sequence, and product.

| **Group** | **SNP** | **Gene** | | **mRNA** | | **Protein** | **GO** | **Annotation** | **Sequence** | **Product** |  |
| --- | --- | --- | --- | --- | --- | --- | --- | --- | --- | --- | --- |
| Group 1 | 101123861\|F\|0-7:C>A-7:C>A | | LOC109504735 | | XM_010941647.3 | XP_010939949.1 | P_GO:0008150 | biological_process | TGCAGGTCAAAAAGATTGTTGAGGGAAGTTTTACAGATCGGAAGAGCGGTTCAGCAGGAATGCCGAGAC | mitochondrial phosphatidate cytidylyltransferase%2C isoform X2 | |
| Group 1 | 101080140\|F\|0-33:G>A-33:G>A | | LOC105059841 | | XM_029260452.1 | XP_029116285.1 | F_GO:0016787 | hydrolase activity | TGCAGTTGGTGACTTGATTTTTATTTATGGAGGGCTACGGTGGTGGTGAGAATTACAGATCGGAAGAGCG | serine/threonine-protein phosphatase BSL3 isoform X1 | |
| Group 1 | 100923387\|F\|0-21:A>G-21:A>G | | LOC105050449 | | XM_010930471.3 | XP_010928773.1 | P_GO:0008150 | biological_process | TGCAGTGATTTTATCATGCTTAGTTTTGGATAGCAGCTTACAGATCGGAAGAGCGGTTCAGCAGGAATG | 26S proteasome non-ATPase regulatory subunit 1 homolog A | |
| Group 1 | 101312759\|F\|0-34:G>A-34:G>A | | LOC105032733 | | XM_010907265.3 | XP_010905567.1 | F_GO:0005515 | protein binding | TGCAGTAGGTTGCAATGGAGGCAAGACCATCAGTGCTGAAGCCATCACAACTGACAAGGGAAAGAGCTT | protein TRANSPORT INHIBITOR RESPONSE 1 | |
| Group 1 | 101312759\|F\|0-34:G>A-34:G>A | | LOC105032211 | | XM_010906601.3 | XP_010904903.2 | F_GO:0005515 | protein binding | TGCAGTAGGTTGCAATGGAGGCAAGACCATCAGTGCTGAAGCCATCACAACTGACAAGGGAAAGAGCTT | vacuolar protein 8 isoform X2 | |
| Group 1 | 101100918\|F\|0-29:C>T-29:C>T | | LOC105050306 | | XM_019852339.2 | XP_019707898.1 | F_GO:0003682 | chromatin binding | TGCAGAAGTTTCTTCATTGGCTGTTACAACTGATCCAGATGTTTCGGCTAAGCTCGACTTTGATTTACA | uncharacterized protein LOC105050306 isoform X2 | |
| Group 1 | 100983116\|F\|0-38:A>G-38:A>G | | LOC105060235 | | XM_010943847.3 | XP_010942149.1 | P_GO:0009987 | cellular process | TGCAGATTTACTCTTTGGAGCAAATAATCCTGTCATCCAAGAAGGAAAAGTATGCTGTTCTTTTCCCTT | aspartate aminotransferase%2C chloroplastic isoform X1 | |
| Group 1 | 101177397\|F\|0-45:C>A-45:C>A | | LOC105033487 | | XM_010908318.3 | XP_010906620.1 | F_GO:0016787 | hydrolase activity | TGCAGTATACTTTTACATTGCAAAGGGAATAGATGGGTGACCTTCACACTCACTTCTGCCATGATGAATT | beta- fructofuranosidase 1 | |
| Group 1 | 100726869\|F\|0-36:G>C-36:G>C | | LOC105041115 | | XM_029263383.1 | XP_029119216.1 | P_GO:0007154 | cell communication | TGCAGTGATGTCAACTGATATGAGAACCAGTATTGTGATGCAAGGAATTACAGATCGGAAGAGCGGTTC | - component response regulator ORR21 isoform | |
| Group 1 | 101058929\|F\|0-37:C>A-37:C>A | | LOC105061372 | | XM_010945397.3 | XP_010943699.1 | F_GO:0003677 | DNA binding | TGCAGGTCAGGTCGTGCTAGACACACCCTTGCCTCTTCGAAGACCTAATACTTGTAAAATGTTGTGCAT | squamosa promoter-binding-like protein 12 | |
| Group 1 | 101176685\|F\|0-52:A>T-52:A>T | | LOC105055198 | | XM_010936950.2 | XP_010935252.1 | P_GO:0006810 | transport | TGCAGGTATGAAAAATCTTTTATCAGGTGGAAGGCAATTGGCACTAACAAGGACAGTGGAAGCATTACA | SEC1 family transport protein SLY1 | |
| Group 1 | 101208530\|F\|0-15:T>C-15:T>C | | LOC105051386 | | XM_010931808.3 | XP_010930110.1 | F_GO:0005488 | binding | TGCAGTTTATGGCTCTCCAGAATCTGCAATAACTAAGGAATGCTTAGAGCATGAATTACAGATCGGAAG | chloroplastic lipoxygenase 6%2C isoform X1 | |
| Group 1 | 101015472\|F\|0-7:T>A-7:T>A | | LOC105049131 | | XM_010928693.3 | XP_010926995.1 | F_GO:0005515 | protein binding | TGCAGGCTGTGATGCAGCAGGAGGTGGAGCAAAAGTTGGCTGTTACAGACAAAAGTGTTACAGATCGGA | protein transport protein SEC31 homolog B isoform X1 | |
| Group 1 | 101288542\|F\|0-46:T>C-46:T>C | | LOC105050554 | | XM_010930628.3 | XP_010928930.1 | P_GO:0009056 | catabolic process | TGCAGAATAGATACTCAGTGCAATTTGATGATTTATTCAAGAAAGCTTGGAGGAAGATTTTACAGATC | DExH -box ATP- dependent RNA helicase DExH11 isoform | |
| Group 1 | 100875773\|F\|0-57:C>T-57:C>T | | LOC105047505 | | XM_010926445.3 | XP_010924747.1 | P_GO:0008150 | biological_process | TGCAGATTCTGCTCCCAGGGACAAATTTCCAAGTTCTTCTGAGAATTCAAAAGGTCCCCGATGCCTCCTT | beta- adaptin -like protein A | |
| Group 1 | 101253873\|F\|0-6:A>G-6:A>G | | LOC105053720 | | XM_019853785.1 | XP_019709344.1 | P_GO:0008152 | metabolic process | TGCAGTAATGGCAACTTGTGATCATGGGTCGGTGGCGAGGATTACAGATCGGAAGAGCGGTTCAGCAGG | H/ACA ribonucleoprotein complex subunit 4 | |
| Group 1 | 101341939\|F\|0-50:C>G-50:C>G | | LOC105055743 | | XM_010937706.3 | XP_010936008.1 | F_GO:0030234 | enzyme regulator activity | TGCAGGATGATGAAAGTTTGAGGAGGTGGAAAGAGAAGCTACTTGGTTGTCTTGAGGGAGAATTACAGA | rho GDP- dissociation inhibitor 1 | |
| Group 1 | 101109738\|F\|0-67:G>A-67:G>A | | LOC105059403 | | XM_010942682.3 | XP_010940984.1 | F_GO:0005488 | binding | TGCAGTAATTGATAACAAACTAATGCCACTTTGAGCCATAATAAGCTCTTGATTGGTTTCTGCAAAGGT | kinesin-like protein KIN-UB isoform X3 | |
| Group 1 | 101142733\|F\|0-21:T>A-21:T>A | | LOC105057024 | | XM_010939456.3 | XP_010937758.1 | F_GO:0003674 | molecular_function | TGCAGCAGCATTCTCTAGCTCTGATCGAAGCCCATATACATGCTCAACAAGTGCTTTCTCTGTAAGAAG | kinesin-like protein KIN-5A | |
| Group 1 | 101143946\|F\|0-5:C>T-5:C>T | | LOC105059180 | | XM_010942412.3 | XP_010940714.1 | F_GO:0003676 | nucleic acid binding | TGCAGCATCCTCAAATTCAACAAAGGCATAGTAACCACCAGAATCCTGCAAGCTCCCAGAAATGCAGTC | putative G3BP-like protein | |
| Group 1 | 101143946\|F\|0-5:C>T-5:C>T | | LOC105059180 | | XM_010942412.3 | XP_010940714.1 | F_GO:0003723 | RNA binding | TGCAGCATCCTCAAATTCAACAAAGGCATAGTAACCACCAGAATCCTGCAAGCTCCCAGAAATGCAGTC | putative G3BP-like protein | |
| Group 1 | 101243149\|F\|0-24:G>A-24:G>A | | LOC105050650 | | XM_010930751.3 | XP_010929053.1 | F_GO:0016740 | transferase activity | TGCAGCTTGTCCAGAGGGTGGTGGGCAATCAATCTTTGAGACAGCATTATTACAGATCGGAAGAGCGGT | E3 ubiquitin -protein ligase UPL1 isoform X4 | |
| Group 1 | 100880174\|F\|0-32:C>T-32:C>T | | LOC105060598 | | XM_010944381.2 | XP_010942683.1 | F_GO:0003677 | DNA binding | TGCAGCTCCTCTATTGACAATCCTTCAAGCTCCTCTCCTCTCATCTGTCTGTAATTGATGGAAAGTATT | MADS-box protein SVP | |
| Group 1 | 101307056\|F\|0-47:G>A-47:G>A | | LOC105037371 | | XM_010913044.2 | XP_010911346.1 | P_GO:0008152 | metabolic process | TGCAGTACCTTCAAAAAACTTTCCACAGCCAGCAATAAACTGTAGAAGAAAAAGAGAGAAAATTACAGA | hydroxyacylglutathione hydrolase cytoplasmic | |
| Group 1 | 101111811\|F\|0-45:T>C-45:T>C | | LOC105045794 | | XM_010924197.3 | XP_010922499.1 | P_GO:0009987 | cellular process | TGCAGTGGATTGTTCCCATGTCTCAAGCATTGTCCGGGGGACTGTTGGATATCTGGACCCTGAGTAAGG | probable LRR receptor-like serine/threonine-protein kinase At1g67720 | |
| Group 1 | 101221362\|F\|0-48:G>A-48:G>A | | LOC105043307 | | XM_019849777.2 | XP_019705336.1 | F_GO:0003723 | RNA binding | TGCAGTTCCCCGGTTTCATTGTAAATGATCCAAATCTTTTAGTGCTCTGTGCATATTTTTTACAGATCG | probable RNA-binding protein 18 isoform | |
| Group 1 | 101221362\|F\|0-48:G>A-48:G>A | | LOC105043307 | | XM_019849777.2 | XP_019705336.1 | F_GO:0003676 | nucleic acid binding | TGCAGTTCCCCGGTTTCATTGTAAATGATCCAAATCTTTTAGTGCTCTGTGCATATTTTTTACAGATCG | probable RNA-binding protein 18 isoform | |
| Group 1 | 100988336\|F\|0-25:G>T-25:G>T | | LOC105053793 | | XM_010935082.3 | XP_010933384.1 | P_GO:0006139 | nucleobase-containing metabolic process | TGCAGGACCTTTTGCAGGCTGTATTGCCATTTGATCTGTTGCTGGATCGAAAAAAGCGTACTAGCTTTC | uncharacterized protein LOC105053793 | |
| Group 1 | 100993448\|F\|0-11:G>C-11:G>C | | LOC105059216 | | XM_010942450.1 | XP_010940752.1 | F_GO:0016787 | hydrolase activity | TGCAGTGACATGCCATCGGCGTTTGGTTTCGGAACGCACCACCTCTTCTCATCCGATGGCATTGGATTC | glucan endo-1%2C3-beta-glucosidase | |
| Group 1 | 100993448\|F\|0-11:G>C-11:G>C | | LOC105059216 | | XM_010942450.1 | XP_010940752.1 | P_GO:0005975 | carbohydrate metabolic process | TGCAGTGACATGCCATCGGCGTTTGGTTTCGGAACGCACCACCTCTTCTCATCCGATGGCATTGGATTC | glucan endo-1%2C3-beta-glucosidase | |
| Group 1 | 100876330\|F\|0-27:C>T-27:C>T | | LOC105058147 | | XM_010940973.3 | XP_010939275.1 | F_GO:0003674 | molecular_function | TGCAGCAAATGAGCGTGTATCTATTGACGAAGAGAACCCAAACTCTACAAGCTTACTCATAAGAGTGTC | pre-mRNA-splicing factor ATP-dependent RNA helicase DEAH1 isoform | |
| Group 1 | 100875830\|F\|0-62:G>A-62:G>A | | LOC105050127 | | XM_010930021.3 | XP_010928323.1 | F_GO:0005515 | protein binding | TGCAGATTGATGACTATAGCTGGCTGGATGGTCATCAATCATCATTCATCAAAAGCATGGAGGTGGAGA | protein SMG7 | |
| Group 1 | 100875830\|F\|0-62:G>A-62:G>A | | LOC105050127 | | XM_010930020.3 | XP_010928322.1 | F_GO:0005515 | protein binding | TGCAGATTGATGACTATAGCTGGCTGGATGGTCATCAATCATCATTCATCAAAAGCATGGAGGTGGAGA | protein SMG7 | |
| Group 2 | 101186201\|F\|0-13:C>T-13:C>T | | LOC105044579 | | XM_010922516.3 | XP_010920818.1 | C_GO:0016020 | membrane | TGCAGTCCTAAAGCCCTGTCTCCTTCAATGCTCTTGAAATCATTACAGATCGGAAGAGCGGTTCAGCAG | chlorophyll synthase%2C chloroplastic | |
| Group 2 | 101183982\|F\|0-6:T>C-6:T>C | | LOC105056931 | | XM_010939309.3 | XP_010937611.1 | C_GO:0005737 | cytoplasm | TGCAGATTCCAGACTTCCACTAGACTCTCCATCTTTCACAGTTATATTCATTACAGATCGGAAGAGCGG | AP3-complex subunit beta-A isoform X2 | |
| Group 2 | 101241427\|F\|0-45:A>C-45:A>C | | LOC105041983 | | XM_019849285.2 | XP_019704844.1 | P_GO:0009987 | cellular process | TGCAGTAAAAAACACATGAACATGATGTACATATCTTCAGCAGTTATTTACAGATCGGAAGAGCGGTTC | 1-phosphatidylinositol-3-phosphate 5-kinase FAB1B isoform | |
| Group 2 | 100924104\|F\|0-14:C>T-14:C>T | | LOC105055686 | | XM_010937606.3 | XP_010935908.1 | P_GO:0008150 | biological_process | TGCAGTTTCCATGGCACCTAGGGTTTTACAGATCGGAAGAGCGGTTCAGCAGGAATGCCGAGACCGATC | structural maintenance of chromosomes protein 1 | |
| Group 2 | 101107315\|F\|0-21:T>C-21:T>C | | LOC105039882 | | XM_019848034.2 | XP_019703593.1 | F_GO:0016301 | kinase activity | TGCAGGACCTCGAGAATGCTCTGACACAACTTCTCCAGTCTCTCGAGATAACTGGAATGGGAGGTATTT | dual specificity protein phosphatase PHS1 isoform X2 | |
| Group 2 | 101120756\|F\|0-40:T>C-40:T>C | | LOC105038521 | | XM_019848695.2 | XP_019704254.1 | F_GO:0003676 | nucleic acid binding | TGCAGAGATTAGCTGGCAATGGAAAACTTAGCAAAAGAGCTATGGGAGTTATTACAGATCGGAAGAGCG | regulator of nonsense transcripts UPF3 | |
| Group 2 | 101014108\|F\|0-38:C>T-38:C>T | | LOC105047396 | | XM_010926314.3 | XP_010924616.1 | F_GO:0003676 | nucleic acid binding | TGCAGCTACTGCATTGGGCAAGCAACACGAGGCCTGACCGGAGTCTTCGAAGGACTTACAGATCGGAAG | polyadenylate-binding protein-interacting protein 9 | |
| Group 2 | 101299938\|F\|0-13:A>G-13:A>G | | LOC105034397 | | XM_010909541.2 | XP_010907843.1 | P_GO:0009987 | cellular process | TGCAGATCCAAGCATAGAAGAATTCCGTTCTGCATTATCTCACACAAAAGATAAAACATCATTCCAAGT | DNA -directed RNA polymerase III subunit 1 | |
| Group 2 | 100986738\|F\|0-20:A>C-20:A>C | | LOC105047854 | | XM_019849453.2 | XP_019705012.1 | F_GO:0005488 | binding | TGCAGCTCAGTTGTGCAATCAATGTCCTCGATCACTAGAATCGATTGGTTCGACATTCGAATCAGCAAT | AAA- ATPase At3g50940 | |
| Group 2 | 100992729\|F\|0-30:T>C-30:T>C | | LOC105059403 | | XM_010942682.3 | XP_010940984.1 | F_GO:0005488 | binding | TGCAGTCATTGATAACAAACTAATGCCACCTTTAGCCATGATTAGCTCTTGATTTGTTTCTGCAAACAT | kinesin-like protein KIN-UB isoform X3 | |
| Group 2 | 101143292\|F\|0-64:T>G-64:T>G | | LOC105056261 | | XM_010938396.3 | XP_010936698.1 | F_GO:0005515 | protein binding | TGCAGCAACAACATATTTCCCCGCAATACCAGGGCACCCTCTTAGAGTATAATGAGACATCACTTCTGT | serine/threonine-protein kinase TOR | |
| Group 2 | 101149036\|F\|0-58:T>A-58:T>A | | LOC105032335 | | XM_010906755.2 | XP_010905057.1 | P_GO:0009058 | biosynthetic process | TGCAGTCTGAAGGCTGGGGGACCAGTTGACCATGTAAGATCCTGAACAATATATTACATAATTTACAGA | valine -- tRNA ligase%2C chloroplastic / mitochondrial 2 | |
| Group 2 | 101160919\|F\|0-19:G>A-19:G>A | | LOC105048658 | | XM_010928048.3 | XP_010926350.1 | F_GO:0005515 | protein binding | TGCAGTTTTCTCGAGCTGAGTCATCTCTTCGATGAAACCTCAAATTTACAGATCGGAAGAGCGGTTCAG | WD and tetratricopeptide repeats protein 1 isoform | |
| Group 2 | 101047813\|F\|0-62:G>T-62:G>T | | LOC105039095 | | XM_010915096.3 | XP_010913398.1 | F_GO:0003824 | catalytic activity | TGCAGAGCAAATTCTGCAATAGGCTTCACATCCCTTGCTCCTTGATAATCTACTGGAGGCTTGCCAGGT | protein disulfide isomerase-like 2-3 | |
| Group 2 | 101017724\|F\|0-12:G>A-12:G>A | | LOC105039171 | | XM_010915213.3 | XP_010913515.1 | C_GO:0005634 | nucleus | TGCAGTTCATATGCCTAATCTGTCTCCATTTACATCTCCCATGTTACAGATCGGAAGAGCGGTTCAGCA | uncharacterized protein LOC105039171 | |
| Group 2 | 101075523\|F\|0-18:T>A-18:T>A | | LOC105051152 | | XM_010931464.3 | XP_010929766.2 | P_GO:0008150 | biological_process | TGCAGCAATTTGATAACGTTCTTTCAAAGAAACTAAATGATGTTGTTACAGATCGGAAGAGCGGTTCAG | protein transport protein Sec24-like At4g32640 | |
| Group 2 | 101322368\|F\|0-40:A>G-40:A>G | | LOC105051390 | | XM_010931823.3 | XP_010930125.1 | P_GO:0008150 | biological_process | TGCAGTCAACTGATGATTTGCCAATCAATATTGGCTTCACAGGCAAGGTATTATTATGTTTTCTTACAG | urease isoform X3 | |
| Group 2 | 101074111\|F\|0-15:C>T-15:C>T | | LOC105046270 | | XM_010924817.3 | XP_010923119.1 | F_GO:0000166 | nucleotide binding | TGCAGACTCAGCAATCGAATGATCAAAGGTTAGGGAGGCTTTACAGATCGGAAGAGCGGTTCAGCAGGA | phosphomethylpyrimidine synthase%2C chloroplastic isoform | |
| Group 2 | 100993389\|F\|0-42:A>G-42:A>G | | LOC105032531 | | XM_029260996.1 | XP_029116829.1 | F_GO:0030234 | enzyme regulator activity | TGCAGTGAAGATCATGAAAACACTTCGCCAACCTGATTTTATACTACCTACCTTTGATTTACAGATCGG | brefeldin A-inhibited guanine nucleotide-exchange protein 5 isoform | |
| Group 2 | 101216940\|F\|0-58:C>T-58:C>T | | LOC105040081 | | XM_019848333.1 | XP_019703892.1 | F_GO:0005515 | protein binding | TGCAGAGCGGTGCCTTTTGTACTCCATGAGGATGTGGGGAGACAGCATGAAGACTCTCCGCTTGGCCTC | uncharacterized protein LOC105040081 isoform X4 | |
| Group 2 | 101171034\|F\|0-15:A>T-15:A>T | | LOC105047908 | | XM_010927039.3 | XP_010925341.1 | P_GO:0000003 | reproduction | TGCAGAAAAAATACTATTCTGTAACTCGTTAGTCACACCGTTTTTTACTGTTTTGGCCATCTTCAAAGA | protein GIGANTEA | |
| Group 2 | 100990555\|F\|0-50:A>T-50:A>T | | LOC105052489 | | XM_010933313.3 | XP_010931615.1 | F_GO:0005515 | protein binding | TGCAGGTCAAGAACATGCAGATCATTGAAGCATGTGGCATGAGAACCCCCACCAAAAATTACAGATCGG | acyl-CoA-binding domain-containing protein 6 | |
| Group 2 | 101052783\|F\|0-63:T>A-63:T>A | | LOC105051336 | | XM_010931710.2 | XP_010930012.2 | C_GO:0016020 | membrane | TGCAGCAAAGCTTGCCAATGCTCATGAATTCATAAGGTACGGGCCAATCAAACGCCAAAAAAATTCAGC | LOW QUALITY PROTEIN: putative multidrug resistance protein | |
| Group 2 | 101160767\|F\|0-18:G>A-18:G>A | | LOC105059704 | | XM_010943122.3 | XP_010941424.1 | P_GO:0006464 | cellular protein modification process | TGCAGTTGTCGAACGCTGGAGGGATCGGGCCGGTTACAGATCGGAAGAGCGGTTCAGCAGGAATGCCGA | leucine-rich repeat receptor-like protein kinase TDR | |
| Group 2 | 100994560\|F\|0-61:T>A-61:T>A | | LOC105043587 | | XM_010921167.1 | XP_010919469.1 | F_GO:0003824 | catalytic activity | TGCAGTTACTATTGATGGTAAACGAAGTGCTCAGTTTGAGCACACTCTTTTGGTTAGTAAATCCATTCA | methionine aminopeptidase 1A isoform X1 | |
| Group 2 | 101195466\|F\|0-65:C>G-65:C>G | | LOC105059431 | | XM_010942723.3 | XP_010941025.1 | P_GO:0008150 | biological_process | TGCAGAAGCCGCTAGCGCACAACATAGTGTCAGGTAAACCTGCATATCAACCCCTCAATGAATTACAGA | low inhibitor 1 | |
| Group 2 | 101102191\|F\|0-44:G>A-44:G>A | | LOC105053424 | | XM_010934564.3 | XP_010932866.1 | P_GO:0008152 | metabolic process | TGCAGAGGAATTTGTCACAATGAGGACTCCAACTCGAAAAATTCGGAGTTCTTCCTCTAGTGCGGTTTG | glutathione reductase%2C chloroplastic | |
| Group 2 | 101108515\|F\|0-45:T>A-45:T>A | | LOC105048281 | | XM_010927545.2 | XP_010925847.1 | F_GO:0000166 | nucleotide binding | TGCAGGGCAGGAAAGATTTCAGAGTCTTGGTGTGGCTTTCTATCGTGGAGCTGATTGCTGTGTTCTCGT | ras-related protein Rab7 | |
| Group 2 | 100919106\|F\|0-34:C>T-34:C>T | | LOC105060509 | | XM_010944247.3 | XP_010942549.1 | F_GO:0016787 | hydrolase activity | TGCAGATCTTCATTTGCAGATGATGTAGTCTGTTCTTTTACAGATCGGAAGAGCGGTTCAGCAGGAATG | 110 kDa U5 small nuclear ribonucleoprotein component CLO | |
| Group 2 | 101048080\|F\|0-44:C>T-44:C>T | | LOC105057588 | | XM_010940235.3 | XP_010938537.1 | P_GO:0006139 | nucleobase-containing compound metabolic process | TGCAGAAAAAGCAGTGCTTTCTCACGAGGGTTTCCTAAATACCGCGGACTTTCCAGGTATGTAATGTTA | AP2-like ethylene-responsive transcription factor At2g41710 | |
| Group 2 | 101142736\|F\|0-33:A>T-33:A>T | | LOC105055849 | | XM_019854315.2 | XP_019709874.1 | F_GO:0003824 | catalytic activity | TGCAGATGCATTTGGAGAACAAGGGAGAGATGCAAGTGATGGATTTCCTGCTATCCCTTCAAATGCTAT | peptidyl-prolyl cis-trans isomerase FKBP62 isoform X2 | |
| Group 2 | 101055908\|F\|0-19:A>G-19:A>G | | LOC105056688 | | XM_029268002.1 | XP_029123835.1 | P_GO:0008152 | metabolic process | TGCAGCTGAGAGTGAACTCAACAAAATCTATGATTGGTCACCTACTTGGAGCTGCTGGTGCAGTGGAAG | 3-oxoacyl-[acyl-carrier-protein] synthase II%2C chloroplastic isoform X5 | |
| Group 2 | 100876085\|F\|0-31:A>G-31:A>G | | LOC105061202 | | XM_010945176.3 | XP_010943478.1 | F_GO:0005215 | transporter activity | TGCAGATTTTTCTTTACAGCATTGCATGGTCATATTGGGTAATTTCTTCTCTAAGCCTGTTACTTTGCA | hippocampus abundant transcript 1 protein | |
| Group 2 | 100873222\|F\|0-6:G>A-6:G>A | | LOC105058794 | | XM_019855157.2 | XP_019710716.1 | F_GO:0005488 | binding | TGCAGAGAAAGCCTCGCCAAATGGGGTCTGACATGACTTGTCTAGCAATACTGAACCAGGTGGCAACAT | alpha-glucan water dikinase%2C chloroplastic isoform | |
| Group 2 | 100994332\|F\|0-60:G>C-60:G>C | | LOC105058527 | | XM_019855006.2 | XP_019710565.2 | F_GO:0005515 | protein binding | TGCAGTGTCCATGAATCAAGACACTATAAACAACCTCATTAGGCTTCCACTTCCTTCTCTAGTATTGATT | pentatricopeptide repeat-containing protein At5g39710 | |
| Group 2 | 101270891\|F\|0-43:G>A-43:G>A | | LOC105054760 | | XM_010936352.3 | XP_010934654.1 | F_GO:0003677 | DNA binding | TGCAGTCCAACTAAAAGCATTCTGTGCTCTTCTTCAGTCCATGGAACTCCTGCAAATCCAACATCATGC | transcription MYBS3 factor | |
| Group 2 | 100888499\|F\|0-19:T>A-19:T>A | | LOC105048553 | | XM_029265542.1 | XP_029121375.1 | P_GO:0019538 | protein metabolism process | TGCAGTGTCTGGGTCTGCATAAGCTGATTTTGCATCCATACAACTGCCATCTCACAATATGAGCACATA | aspartic proteinase oryzasin-1 | |
| Group 2 | 100995993\|F\|0-58:G>T-58:G>T | | LOC105056325 | | XM_019854501.2 | XP_019710060.1 | P_GO:0008152 | metabolic process | TGCAGTTTTTTCACTCTTTACTGGCTTATATAAATGGTAAGCATAACAACAAAGTCTAGCTTGGATTAG | calpain-type cysteine protease ADL1 | |
| Group 2 | 100887191\|F\|0-22:T>A-22:T>A | | LOC105057265 | | XM_010939820.3 | XP_010938122.1 | P_GO:0009987 | cellular process | TGCAGTCTAATGGGGCAATCAATGCACAGAATGTCCCAGATTTTTCAGGAGATTCAGCTAGCCTGCCTA | kinesin-like protein KIN-14B isoform X1 | |
| Group 2 | 100980803\|F\|0-39:C>T-39:C>T | | LOC105032615 | | XM_019846349.2 | XP_019701908.1 | F_GO:0016787 | hydrolase activity | TGCAGAGAAGAAACCAAAATTCATCATGTAATAATGAAGCCTGTTTTTTCGATCATCGTCAAGGTTAGT | serine/threonine-protein phosphatase PP1 | |
| Group 2 | 101061297\|F\|0-9:T>A-9:T>A | | LOC105037238 | | XM_010912929.3 | XP_010911231.2 | F_GO:0005198 | structural molecule activity | TGCAGTCTATGAAATAAGCTCACCTGCACTGGCCGATGATAACATGGTCACCCTCTTTCACACGGAAGC | 40S ribosomal protein S11-1 | |
| Group 2 | 101110590\|F\|0-28:T>G-28:T>G | | LOC105043469 | | XM_010921024.2 | XP_010919326.1 | F_GO:0003677 | DNA binding | TGCAGTCACTTTCGCATATTGTGGATGCTTCCATGGAAGATTTGGCTCGTTGCCCGGGCATTGGTGAAC | DNA excision repair protein ERCC-1 | |
| Group 2 | 100989757\|F\|0-14:A>T-14:A>T | | LOC105056273 | | XM_029267907.1 | XP_029123740.1 | P_GO:0008150 | biological_process | TGCAGGGAATTGGCATATGCTTTATTTGATACTTTGTCAAGAAAACTTCAATATGCATCCAGGAGTAAG | serine/threonine-protein kinase ATM isoform X4 | |
| Group 2 | 100882810\|F\|0-43:A>T-43:A>T | | LOC105054473 | | XM_010935992.3 | XP_010934294.1 | F_GO:0016301 | kinase activity | TGCAGGCATACTCTACCGTTGGTACACCTGATTATATTGCTCCAGAAGTTTTACTGAAGAAAGGTTATG | serine/threonine-protein kinase tricorner isoform | |
| Group 2 | 100882810\|F\|0-43:A>T-43:A>T | | LOC105033607 | | XM_029261374.1 | XP_029117207.1 | F_GO:0016740 | transferase activity | TGCAGGCATACTCTACCGTTGGTACACCTGATTATATTGCTCCAGAAGTTTTACTGAAGAAAGGTTATG | LOW QUALITY PROTEIN: serine/threonine-protein kinase tricorner | |
| Group 2 | 100886264\|F\|0-14:G>C-14:G>C | | LOC105056325 | | XM_019854501.2 | XP_019710060.1 | P_GO:0008152 | metabolic process | TGCAGTATAGCCCTGAAGTACAGATACAGGTGCTATGGTGGAACGAGAGCAAAGCTGTTGCATTACCTT | calpain-type cysteine protease ADL1 | |
| Group 2 | 100882252\|F\|0-63:G>T-63:G>T | | LOC105055908 | | XM_029267822.1 | XP_029123655.1 | P_GO:0008152 | metabolic process | TGCAGGATATGGCAAAATGCCAGGCTATGAACACATTTTCACTGACTTCCTCCTAAGCCTGGTGCAAAG | inositol- tetrakisphosphate 1-kinase 3 isoform | |
| Group 2 | 100877705\|F\|0-29:G>A-29:G>A | | LOC105047460 | | XM_029265258.1 | XP_029121091.1 | P_GO:0005975 | carbohydrate metabolic process | TGCAGCATATCCCTTGCCTGAAGCCTTCCGAATTCAACATCTGCCACAATAACAAGAAGAAAAGGTTGT | alpha%2Calpha-trehalose-phosphate synthase [UDP-forming] 1 isoform | |
| Group 2 | 101056922\|F\|0-66:A>C-66:A>C | | LOC105060113 | | XM_029260477.1 | XP_029116310.1 | F_GO:0005515 | protein binding | TGCAGGACCATCTTTAGGAGATTTTGTTCTTTGGATGGGGGATCAAGCAAACTCCACACCTGCTCCAGC | uncharacterized protein LOC105060113 isoform X3 | |
| Group 3 | 101050938\|F\|0-8:G>A-8:G>A | | LOC105040322 | | XM_029263142.1 | XP_029118975.1 | F_GO:0003677 | DNA binding | TGCAGAGGGAGGTACAGTGTTGAGCTTCACCTTTCATTCTTGCGACTGCAAGGACAGGCTAATGATTTT | FACT complex subunit SSRP1-A isoform X2 | |
| Group 3 | 101201805\|F\|0-45:T>G-45:T>G | | LOC105042309 | | XM_010919460.2 | XP_010917762.1 | P_GO:0009987 | cellular process | TGCAGTTGCTGCTAATGTTGCCTCTTCGTCTGCAACATCTAAAACTTCCCTGCAACTTTTTACAGATCGG | tRNA ( guanine -N(7)-)- non- catalytic methyltransferase wdr4 isoform X1 subunit | |
| Group 3 | 101149179\|F\|0-47:C>T-47:C>T | | LOC105047991 | | XM_019846402.2 | XP_019701961.1 | C_GO:0005737 | cytoplasm | TGCAGTGAACACACTTGTTACATTGGGAAGTGAGAATGCATTATCATCGATAAATAGGTCATAATTTGT | protein farnesyltransferase subunit beta isoform X1 | |
| Group 3 | 101330977\|F\|0-46:C>T-46:C>T | | LOC105051986 | | XM_010932657.3 | XP_010930959.1 | F_GO:0005515 | protein binding | TGCAGTCAAATGCACGTACTGACCGATCATAGCCAGCCGACACAACCACCGAATCATACTCATTACAGA | WD repeat domain-containing protein 83 | |
| Group 3 | 100723150\|F\|0-16:A>G-16:A>G | | LOC105035991 | | XM_029262107.1 | XP_029117940.1 | F_GO:0000166 | nucleotide binding | TGCAGAGCACAGAACAACAGGTTCAAGGAGTGTTGAATTTACAGATCGGAAGAGCGGTTCAGCAGGAAT | kinesin-like protein KIN-14H isoform X4 | |
| Group 3 | 101279639\|F\|0-44:C>G-44:C>G | | LOC105047935 | | XM_019849297.2 | XP_019704856.1 | P_GO:0006464 | cellular protein modification process | TGCAGTGTTGAAGAGGATATGGTGGATGGTTTCCCATATGAAGTCCCAGAGGAATACAGCAGTATGCCT | peptidyl-prolyl cis-trans isomerase CYP38%2C chloroplastic isoform X1 | |
| Group 3 | 101294328\|F\|0-51:G>T-51:G>T | | LOC105034143 | | XM_010909191.3 | XP_010907493.2 | F_GO:0005515 | protein binding | TGCAGTCTTGGACATGTATGCAAAATGTGGAGAATTGGATATCGCAGAGCAGATCTTTGATGAGATTTT | putative pentatricopeptide repeat-containing protein At3g11460%2C mitochondrial | |
| Group 3 | 101075145\|F\|0-9:T>A-9:T>A | | LOC105047601 | | XM_010926592.3 | XP_010924894.1 | P_GO:0008150 | biological_process | TGCAGATTCTGACATCCCTTGCTTTTTATTTTATTACAGATCGGAAGAGCGGTTCAGCAGGAATGCCGAG | importin beta-1 subunit | |
| Group 3 | 101185570\|F\|0-24:A>G-24:A>G | | LOC105061468 | | XM_019846088.2 | XP_019701647.1 | P_GO:0008150 | biological_process | TGCAGGTAACTGGCCTTTAGGCCCACTCTGCAAGGATGGTTTCCACAAAAGATTACAGATCGGAAGAGC | kinesin-like protein KIN-5C isoform X1 | |
| Group 3 | 101159783\|F\|0-11:G>A-11:G>A | | LOC105042667 | | XM_019849750.2 | XP_019705309.1 | F_GO:0005515 | protein binding | TGCAGTCACATGTGCATTACCACAGCCAATGGCAGAAAGCAAGAGGTCAGTTCTTTTACAGATCGGAAG | uncharacterized protein LOC105042667 isoform X4 | |
| Group 3 | 101186789\|F\|0-34:C>T-34:C>T | | LOC105053183 | | XM_029267043.1 | XP_029122876.1 | F_GO:0003677 | DNA binding | TGCAGTTTCATAATAATTTCCATGTACGAAATCACAATATAGACAGGGGATCCTTTACAGATCGGAAGA | DNA repair protein RAD4 isoform X2 | |
| Group 3 | 101186789\|F\|0-34:C>T-34:C>T | | LOC105053183 | | XM_010934254.3 | XP_010932556.1 | F_GO:0003677 | DNA binding | TGCAGTTTCATAATAATTTCCATGTACGAAATCACAATATAGACAGGGGATCCTTTACAGATCGGAAGA | DNA repair protein RAD4 isoform X1 | |
| Group 3 | 101186789\|F\|0-34:C>T-34:C>T | | LOC105053183 | | XM_010934255.3 | XP_010932557.1 | F_GO:0003677 | DNA binding | TGCAGTTTCATAATAATTTCCATGTACGAAATCACAATATAGACAGGGGATCCTTTACAGATCGGAAGA | DNA repair protein RAD4 isoform X3 | |
| Group 3 | 101317305\|F\|0-6:G>A-6:G>A | | LOC105053578 | | XM_010934792.3 | XP_010933094.1 | C_GO:0005634 | nucleus | TGCAGAGCTACGGGAGATGCTGGAAGCTAATGGGCAGGATTCAGCAGGATCAGAATATGATCTACGAGA | LOW QUALITY PROTEIN: poly [ADP-ribose] polymerase 1-like | |
| Group 3 | 101109523\|F\|0-32:A>C-32:A>C | | LOC105055389 | | XM_010937177.2 | XP_010935479.1 | F_GO:0016787 | hydrolase activity | TGCAGTAAAACGGGAACTAGACCTTACCGGAGACAATCCATCAGCTGGTTCTATGCTGATGTCAAAAG | likely cytosolic oligopeptidase A | |
| Group 3 | 101241030\|F\|0-30:G>T-30:G>T | | LOC105055234 | | XM_010937002.3 | XP_010935304.2 | F_GO:0003824 | catalytic activity | TGCAGCGCAAGTTGATAATCAAGTGCATGAGAGCCATTTTCAGAGAAAAAATAATTACAGATCGGAAGA | Fanconi anemia group J protein homolog isoform X2 | |
| Group 3 | 101144970\|F\|0-15:A>T-15:A>T | | LOC105033907 | | XM_029261435.1 | XP_029117268.1 | F_GO:0003676 | nucleic acid binding | TGCAGCTAACCCCTAAGCAACCGATGCCACAGCCGGACACTGGTAATAGGATCGATGAACCAAACATTA | zinc finger CCCH domain-containing protein 18 isoform | |
| Group 3 | 101219203\|F\|0-14:C>A-14:C>A | | LOC105042690 | | XM_029263874.1 | XP_029119707.1 | F_GO:0005488 | binding | TGCAGGAGAAAAGGCGAGCTCTTATTGGTCTATGAATTCATGCCCAATGGTAGTTTGGATAAGTTCCTT | L-type lectin-domain containing receptor kinase IV.1-like isoform X2 | |
| Group 3 | 100992404\|F\|0-39:G>A-39:G>A | | LOC105038140 | | XM_010913852.2 | XP_010912154.1 | F_GO:0005515 | protein binding | TGCAGTATTTACTCTCTTCTGGATCTTGCCAAGGAGGCAGGAGCAAATTTACAGCCTTTGTTTCAATTT | protein ROOT INITIATION DEFECTIVE 3 isoform | |
| Group 3 | 101308012\|F\|0-43:A>G-43:A>G | | LOC105041846 | | XM_019849284.2 | XP_019704843.1 | F_GO:0005515 | protein binding | TGCAGTATCTTTACAATGTGCTTGGAGACAGAAACTGGCAAGAAGAGAACTTCGGAAGCTTACAGATCG | LOW QUALITY PROTEIN: OPAQUE1-like protein | |
| Group 3 | 101062304\|F\|0-48:G>A-48:G>A | | LOC105037169 | | XM_010912864.3 | XP_010911166.1 | F_GO:0005515 | protein binding | TGCAGTGTACAAGAAGTGGACAAAGTACCGAAGAACATCATATGAGACGTCATGGATCTTACAGATCGG | BTB/POZ domain-containing protein At4g08455 | |
| Group 3 | 100982962\|F\|0-48:G>A-48:G>A | | LOC105050104 | | XM_010929974.3 | XP_010928276.1 | F_GO:0005515 | protein binding | TGCAGATTCTTCAGCCCACTTTAGCTCTTCTGATGCAGTAATAGTCACGAGGTCACCTTCTTTGTCCTT | LOW QUALITY PROTEIN: PHOX1-like protein | |
| Group 3 | 100982962\|F\|0-48:G>A-48:G>A | | LOC105039069 | | XM_010915053.3 | XP_010913355.1 | F_GO:0005515 | protein binding | TGCAGATTCTTCAGCCCACTTTAGCTCTTCTGATGCAGTAATAGTCACGAGGTCACCTTCTTTGTCCTT | protein PHOX1 | |
| Group 3 | 101183113\|F\|0-24:G>A-24:G>A | | LOC105045901 | | XM_010924339.2 | XP_010922641.1 | P_GO:0009058 | biosynthetic process | TGCAGAACCTGTTATGAGTATGGTGAAGACTGAGGCAGATGAGGAGCTTTTTACAGATCGGAAGAGCGG | dehydration-responsive element-binding protein 2C isoform | |
| Group 3 | 101198012\|F\|0-65:A>T-65:A>T | | LOC105045102 | | XM_010923262.3 | XP_010921564.1 | F_GO:0005515 | protein binding | TGCAGCCCCTTGTTGAGGAAGGACGGGCTAAACATCTCCCATGTCCCAAATATTTTTATTGATCAAGGT | putative disease resistance protein RGA1 | |
| Group 3 | 100993674\|F\|0-63:C>T-63:C>T | | LOC105058237 | | XM_010941113.3 | XP_010939415.1 | F_GO:0003824 | catalytic activity | TGCAGTGCAACGTGGCCTTCCGGTTACAAGAGTTATTAGCCGTATGACTTTGCAAGAAATATTCGCCCG | chloroplastic zeaxanthin epoxidase%2C | |
| Group 3 | 100993586\|F\|0-22:C>T-22:C>T | | LOC105042639 | | XM_010919933.3 | XP_010918235.1 | F_GO:0005515 | protein binding | TGCAGTGATCGATGATCTTGTTCGTGCTACTATTGCTGGATTGGAGAAGGGAAAACCGCCTGTCATGTC | phosphatidylinositol 4-kinase gamma 4 | |
| Group 3 | 100993586\|F\|0-22:C>T-22:C>T | | LOC105042639 | | XM_010919926.3 | XP_010918228.1 | F_GO:0005515 | protein binding | TGCAGTGATCGATGATCTTGTTCGTGCTACTATTGCTGGATTGGAGAAGGGAAAACCGCCTGTCATGTC | phosphatidylinositol 4-kinase gamma 4 | |
| Group 3 | 101252932\|F\|0-22:A>T-22:A>T | | LOC105055686 | | XM_010937606.3 | XP_010935908.1 | P_GO:0008150 | biological_process | TGCAGAAATTGCTGAAGAAAGAACACTTTCTGTGGCAACTTTTTACAGATCGGAAGAGCGGTTCAGCAG | structural maintenance of chromosomes protein 1 | |
| Group 3 | 101174882\|F\|0-63:T>A-63:T>A | | LOC105052814 | | XM_029266945.1 | XP_029122778.1 | F_GO:0005488 | binding | TGCAGCTAATTGTTGCCTTGCTACCATGTCTTCTGTGAATATCTCCTTCAGCAATACAAGTAATAAGTT | transcription factor SPATULA isoform X3 | |
| Group 3 | 100986946\|F\|0-55:C>T-55:C>T | | LOC105048612 | | XM_010927965.3 | XP_010926267.1 | P_GO:0009987 | cellular process | TGCAGCTCTTCAACTGTCTCGATGTTGTGAGCAAAAACATCTAACCCGGATTTTGCAACTGTCTCCACG | mitochondrial lipoyl synthase%2C | |
| Group 3 | 100986946\|F\|0-55:C>T-55:C>T | | LOC105048612 | | XM_010927965.3 | XP_010926267.1 | P_GO:0008150 | biological_process | TGCAGCTCTTCAACTGTCTCGATGTTGTGAGCAAAAACATCTAACCCGGATTTTGCAACTGTCTCCACG | mitochondrial lipoyl synthase%2C | |
| Group 3 | 100638248\|F\|0-11:C>G-11:C>G | | LOC105033711 | | XM_010908612.3 | XP_010906914.1 | F_GO:0003677 | DNA binding | TGCAGCCTTGTCTTGGCAATTGCTGTGGTTATGGACCCTCGTTTCAAGTTGAAGCTTGTGGAGTTCAGT | zinc finger BED domain-containing protein RICESLEEPER 2 | |
| Group 3 | 101059433\|F\|0-43:A>C-43:A>C | | LOC105044682 | | XM_010922672.3 | XP_010920974.2 | F_GO:0005488 | binding | TGCAGGTTTCAAGATCTATTGGGGATGCTTATTTGAAGGATGCAGAGTTCAACCGTGAGCCTCTACTAT | probable protein phosphatase 2C 38 | |
| Group 3 | 101145495\|F\|0-41:A>T-41:A>T | | LOC105053654 | | XM_010934907.3 | XP_010933209.1 | P_GO:0008150 | biological_process | TGCAGCTTCTCCAACTCCTTTGATGCATAGAGAAGAGCAACACCACCACCTACAAAGAACAGAATGATA | mitochondrial chaperonin CPN60-2%2C | |
| Group 3 | 100642518\|F\|0-13:C>A-13:C>A | | LOC105042450 | | XM_029263819.1 | XP_029119652.1 | F_GO:0016740 | transferase activity | TGCAGGGAACTGGCAAGAAGAAAATGGAGCACCAGCTTGAGCAGTTAGAAGTGAAGTATCCAGACAAGG | granule-bound starch synthase 1b%2C chloroplastic /amyloplastic isoform | |
| Group 3 | 100642518\|F\|0-13:C>A-13:C>A | | LOC105042450 | | XM_010919674.3 | XP_010917976.1 | F_GO:0016740 | transferase activity | TGCAGGGAACTGGCAAGAAGAAAATGGAGCACCAGCTTGAGCAGTTAGAAGTGAAGTATCCAGACAAGG | granule-bound starch synthase 1b%2C chloroplastic /amyloplastic isoform | |
| Group 4 | 101294238\|F\|0-12:T>C-12:T>C | | LOC105050760 | | XM_010930899.2 | XP_010929201.1 | F_GO:0003824 | catalytic activity | TGCAGTATCAAGTGCTGATGATCTTGATGACAAATCAAAAGCTCCACTTGTGCAGCAAAGGGGACGTTT | serine/threonine-protein kinase 4 isoform | |
| Group 4 | 101294238\|F\|0-12:T>C-12:T>C | | LOC105050760 | | XM_019852149.2 | XP_019707708.1 | F_GO:0003824 | catalytic activity | TGCAGTATCAAGTGCTGATGATCTTGATGACAAATCAAAAGCTCCACTTGTGCAGCAAAGGGGACGTTT | serine/threonine-protein kinase 4 homolog B isoform X3 | |
| Group 4 | 101278166\|F\|0-54:T>G-54:T>G | | LOC105047665 | | XM_010926698.3 | XP_010925000.3 | F_GO:0005488 | binding | TGCAGATATGGGATTCTGTTCCAAAACCACAAGCTCACAAACAAACTCCACTTATTGAATTTTTTACAG | LOW QUALITY PROTEIN: protein ROOT HAIR DEFECTIVE 3 | |
| Group 4 | 101278166\|F\|0-54:T>G-54:T>G | | LOC105047665 | | XM_010926698.3 | XP_010925000.3 | F_GO:0000166 | nucleotide binding | TGCAGATATGGGATTCTGTTCCAAAACCACAAGCTCACAAACAAACTCCACTTATTGAATTTTTTACAG | LOW QUALITY PROTEIN: protein ROOT HAIR DEFECTIVE 3 | |
| Group 4 | 100918738\|F\|0-14:G>A-14:G>A | | LOC105039669 | | XM_010915896.3 | XP_010914198.2 | F_GO:0005515 | protein binding | TGCAGAGGGAGGGTGGAGGAGGCTTACCAGTGTTTTACAGATCGGAAGAGCGGTTCAGCAGGAATGCCG | pentatricopeptide repeat-containing protein At3g61360 | |
| Group 4 | 100884276\|F\|0-5:G>A-5:G>A | | LOC105045376 | | XM_010923637.3 | XP_010921939.1 | P_GO:0008150 | biological_process | TGCAGGTAATTGGCTTGAGCAGTGTTCCTTTTGCTGGTGATGAATTTGAGGTTGTTGACTCCCTTGATG | translation initiation factor IF-2%2C chloroplastic isoform X2 | |
| Group 4 | 101059415\|F\|0-28:G>A-28:G>A | | LOC105058896 | | XM_029260259.1 | XP_029116092.1 | F_GO:0003676 | nucleic acid binding | TGCAGGTTGTAGCTTCTCAAGATTGGCCGGAAGTAACAAAGTATGCTGGATTAGTTTCTGCTCAGGCCC | protein argonaute 1B | |
| Group 4 | 100629405\|F\|0-29:A>T-29:A>T | | LOC105056685 | | XM_010938979.3 | XP_010937281.1 | F_GO:0016787 | hydrolase activity | TGCAGGACATTCTCTTCCAGAGATATCAGAGTTCTCTGTTTTATCTTCTACAGGTCCATTTACAGAATT | phosphoinositide phosphatase SAC2 | |
| Group 4 | 101049061\|F\|0-63:C>T-63:C>T | | LOC105034902 | | XM_019847131.2 | XP_019702690.1 | P_GO:0006810 | transport | TGCAGAAGCATCTGAAAGGGTTTCATGGCTTGCAATTGCATGGGATCGGAGAGTCCAAGTTGCCAAGTT | vacuolar protein sorting-associated protein 8 homolog isoform X5 | |
| Group 4 | 101109631\|F\|0-19:C>T-19:C>T | | LOC105050163 | | XM_010930073.3 | XP_010928375.1 | F_GO:0016787 | hydrolase activity | TGCAGTAACTATGTCCAGTCCCTTCTTGGAAGCAAGGTATGGAGCATTCTGCAAACATACGATGATTGG | LOW QUALITY PROTEIN: glycerophosphodiester phosphodiesterase GDPDL7-like | |
| Group 4 | 101217435\|F\|0-27:T>A-27:T>A | | LOC105061433 | | XM_010945475.3 | XP_010943777.1 | F_GO:0003674 | molecular_function | TGCAGATGCAGGTGTCATACCAAGAGCTGTCAAACAGATTTTTGACACATTAGAAAGTCAAAATGCTGA | kinesin-like protein KIN-5C isoform X2 | |
| Group 4 | 101226190\|F\|0-23:C>T-23:C>T | | LOC105045331 | | XM_010923573.3 | XP_010921875.1 | P_GO:0008150 | biological_process | TGCAGGGAAGTTTCTACACAGGCCATTCTTCAAGCATTACAGATCGGAAGAGCGGTTCAGCAGGAATGC | probable glycerol-3-phosphate dehydrogenase [ NAD( +)] 1%2C cytosolic | |
| Group 4 | 100980678\|F\|0-15:T>C-15:T>C | | LOC105046576 | | XM_010925199.3 | XP_010923501.1 | P_GO:0008152 | metabolic process | TGCAGACTTGACAAATGAAACTGAAATAAAATCCACACCCTCAGCAATCCCAAAGTCTATGTCCAGCCA | pyruvate kinase isozyme A%2C chloroplastic | |
| Group 4 | 101176367\|F\|0-27:G>T-27:G>T | | LOC105057103 | | XM_010939559.3 | XP_010937861.1 | P_GO:0008150 | biological_process | TGCAGGGACAGTGGATCACCTCATGTTGATAGACTAATGGGCCATGGGGATGTGGAGGTTCAGTCATGT | metal tolerance protein 1 | |
| Group 4 | 101174807\|F\|0-21:C>T-21:C>T | | LOC105058945 | | XM_010942032.3 | XP_010940334.1 | F_GO:0003674 | molecular_function | TGCAGCGTAGCCTTGGCCCTTCACTTGTGATATGAAGGTGGAGATTGTTCGGCCCAAAACCTTACAGAT | probable metal- nicotianamine transporter YSL12 | |
| Group 4 | 101159720\|F\|0-8:T>A-8:T>A | | LOC105051364 | | XM_010931757.3 | XP_010930059.1 | F_GO:0005515 | protein binding | TGCAGTATTCTGTGTTCTTATGTGGTCTGTTCCATTACAGATCGGAAGAGCGGTTCAGCAGGAATGCCG | serine/threonine-protein phosphatase 6 regulatory ankyrin repeat subunit B | |
| Group 4 | 101061183\|F\|0-57:T>C-57:T>C | | LOC105035665 | | XM_029262015.1 | XP_029117848.1 | F_GO:0003824 | catalytic activity | TGCAGTCGCAATCAGATATTTGTCATCCCCTTCACCTGTCACCTTTCAAAGATCAACTAATATCACAAT | serine -- tRNA ligase isoform X1 | |
| Group 4 | 100981397\|F\|0-12:C>A-12:C>A | | LOC105041758 | | XM_029263808.1 | XP_029119641.1 | F_GO:0003824 | catalytic activity | TGCAGAGGATGGCGAGTGTCATCTTCATCTGGAGATAAGCAGAATCCTTCCCCAAACATATCCGAGGTC | cytochrome P450 704B1 | |
| Group 4 | 100634510\|F\|0-32:C>T-32:C>T | | LOC105059999 | | XM_010943541.3 | XP_010941843.1 | F_GO:0003677 | DNA binding | TGCAGATGGCTCTTCACTTCATCATTGGTAAGCCCATCGACCTTCATCAGTTCTCTGATCTGCTTTGGA | myb family transcription factor EFM | |
| Group 4 | 101233977\|F\|0-30:G>A-30:G>A | | LOC105056876 | | XM_019854549.2 | XP_019710108.1 | P_GO:0005975 | carbohydrate metabolic process | TGCAGACATAGCATATTTTTGCACGTAGCGCCACAATTCACATCCATTCCTGAAATTTTACAGATCGG | probable beta-D- xylosidase 7 | |
| Group 4 | 101057748\|F\|0-25:G>T-25:G>T | | LOC105049304 | | XM_029265787.1 | XP_029121620.1 | F_GO:0005488 | binding | TGCAGGCCACCGCCGGGATGTCATCGCTGATGAACATGGCCCCACAGAAGGCTGTTGTAGCGGAAACTG | putative inactive cadmium / zinc-transporting HMA3 ATPase | |
| Group 5 | 101157871\|F\|0-48:C>A-48:C>A | | LOC105051547 | | XM_010932046.3 | XP_010930348.3 | F_GO:0005488 | binding | TGCAGCCATTTTGGAGTGAACCAGCTATCTGGTACCATCCCAAGTAAACTTTTTACAGATCGGAAGAGC | probable leucine-rich repeat receptor-like protein kinase At5g49770 | |
| Group 5 | 101176795\|F\|0-43:A>G-43:A>G | | LOC105060633 | | XM_029260596.1 | XP_029116429.1 | F_GO:0005515 | protein binding | TGCAGGTGAAAACCAAGCTCAAGGGTCACCAGAAGAAGATCACAGGCCTTGCATTTTCCCAGTCATTAC | protein TOPLESS-RELATED PROTEIN 2 isoform | |
| Group 5 | 101013087\|F\|0-13:C>T-13:C>T | | LOC105056458 | | XM_010938663.3 | XP_010936965.1 | P_GO:0009987 | cellular process | TGCAGCACATCAACGCCATTAGAAAGTCACCTGTTGGTGCACAAATTACAGATCGGAAGAGCGGTTCAG | transcription initiation factor IIE subunit beta | |
| Group 5 | 100977892\|F\|0-48:T>C-48:T>C | | LOC105036108 | | XM_010911848.3 | XP_010910150.1 | P_GO:0008150 | biological_process | TGCAGAAAATTCTGGCCCAGGGTCATTGGGTTCTGACTCAGAATCATTTGCAGCTTGTTTTACAGATCG | translocase of chloroplast 159%2C chloroplastic | |
| Group 5 | 101015285\|F\|0-9:A>T-9:A>T | | LOC105034020 | | XM_010909041.3 | XP_010907343.2 | P_GO:0005975 | carbohydrate metabolic process | TGCAGGCATATCTTTTCAATGATTACTGGGAGGATATTGGGACTATTACAGATCGGAAGAGCGGTTCAG | glucose-1-phosphate adenylyltransferase large subunit 1%2C chloroplastic | |
| Group 5 | 101125543\|F\|0-51:A>C-51:A>C | | LOC105049419 | | XM_019852391.1 | XP_019707950.1 | F_GO:0005515 | protein binding | TGCAGTTCCTTGATGAGGAGAAGTTCAAAGAGACTGTTCACAAGTGCGTTTACTTGTTACAGATCGGAA | TPR3 protein | |
| Group 5 | 101012350\|F\|0-7:C>T-7:C>T | | LOC105054038 | | XM_019846676.2 | XP_019702235.1 | F_GO:0005515 | protein binding | TGCAGATCCTTAGAGAGGATCTTGCTTCTTTACAGATCGGAAGAGCGGTTCAGCAGGAATGCCGAGACC | uncharacterized protein LOC105054038 isoform X2 | |
| Group 5 | 101157534\|F\|0-37:C>T-37:C>T | | LOC105046359 | | XM_010924928.2 | XP_010923230.1 | P_GO:0008152 | metabolic process | TGCAGCAGCAATCTCCAAGGATGCCAGGGCCTGCACTCCAAAAGTCTTTACAGATCGGAAGAGCGGTTC | transcription initiation factor TFIID subunit 12b isoform X2 | |
| Group 5 | 100919347\|F\|0-18:C>T-18:C>T | | LOC105040517 | | XM_029263204.1 | XP_029119037.1 | P_GO:0008150 | biological_process | TGCAGATTCTGAGGATCTCCGATTATGGAAGGGATGGGTTGAATCTCGATTACAGATCGGAAGAGCGGT | nuclear poly(A) polymerase 4 isoform X4 | |
| Group 5 | 100990740\|F\|0-33:A>G-33:A>G | | LOC105043997 | | XM_029264213.1 | XP_029120046.1 | F_GO:0005515 | protein binding | TGCAGGTGAAATTCTATCCGGGCGCTCAAGGGTAGCAGCTAGAGAACCAGAAACATTTGAAACAGCACC | protein TOPLESS-RELATED PROTEIN 2 isoform | |
| Group 5 | 101150885\|F\|0-45:C>G-45:C>G | | LOC105043492 | | XM_010921052.3 | XP_010919354.1 | F_GO:0016301 | kinase activity | TGCAGTTTTGGAACTCTCTGAGAAATATCAGAAACGTTTTGGTCCCTTACGGTACTTCGTGGCTGGTTT | sphingoid long-chain bases kinase 1 isoform | |
| Group 5 | 101341450\|F\|0-62:G>C-62:G>C | | LOC105055845 | | XM_029267809.1 | XP_029123642.1 | F_GO:0003824 | catalytic activity | TGCAGATAATTTGCACATTGTTCATTTCTAGGACTAGCATATTCTCCTCCACAACTATTCTTGGTTGAC | serine carboxypeptidase-like 18 isoform X6 | |
| Group 5 | 100985719\|F\|0-41:C>A-41:C>A | | LOC105053892 | | XM_010935226.3 | XP_010933528.1 | F_GO:0016787 | hydrolase activity | TGCAGCCTATAGGATCTTCCTTCAAGAACCTTTATAGCAAGCAATAATGGGATTAGCAATGGTAAACAA | dynamin-related protein 1E | |
| Group 5 | 100645251\|F\|0-31:A>T-31:A>T | | LOC105047114 | | XM_010925924.3 | XP_010924226.1 | P_GO:0008150 | biological_process | TGCAGTATCTGCATACAGAGATTCTCTATATATTCTTCCTCCATCTTCAGCAAAACTTCCCTGACAAAA | T-complex protein 1 subunit gamma | |
| Group 5 | 101012888\|F\|0-36:C>G-36:C>G | | LOC105049848 | | XM_010929619.3 | XP_010927921.1 | F_GO:0005515 | protein binding | TGCAGCAACATACAACGCTGTCTCTCCATCATGGTTCTGCTTAGAAATCAAATCTTTTACAGATCGGAA | ankyrin repeat-containing protein At5g02620 | |
| Group 5 | 101079801\|F\|0-34:G>A-34:G>A | | LOC105061376 | | XM_029260737.1 | XP_029116570.1 | F_GO:0005515 | protein binding | TGCAGTTACTGGTGGTATTGCATGACATGGTAGAGAAGGGGCAGAGTTATCTGATTACAGATCGGAAGA | pentatricopeptide repeat-containing protein At5g66520-like | |
| Group 5 | 101107014\|F\|0-14:G>C-14:G>C | | LOC105054277 | | XM_010935769.3 | XP_010934071.1 | F_GO:0003824 | catalytic activity | TGCAGGAAATTGATGAACTTTGTGAGGAGTGGGTCCCAGAATCTCTTCATCCACCTATCACCGAAGAGA | long chain base biosynthesis protein 1b | |
| Group 5 | 101253100\|F\|0-19:G>A-19:G>A | | LOC105050009 | | XM_019851804.2 | XP_019707363.1 | P_GO:0009987 | cellular process | TGCAGAGGGTCTTTTCTCTGTCAAGTCTGATGTCTTTAGCTTTGGAGTCTTGCTTTTAGAGATCGGAAG | G-type lectin S-receptor-like serine/threonine-protein kinase At1g11300 | |
| Group 5 | 100989400\|F\|0-65:A>T-65:A>T | | LOC105033409 | | XM_029261307.1 | XP_029117140.1 | F_GO:0003824 | catalytic activity | TGCAGGCGAAAATCTTCTTCATGAAAGAACTTGGACAAGTCAGTGTGGAATGTTACTTTGAATTTATGC | serine/threonine-protein kinase GRIK1 isoform X3 | |
| Group 5 | 100634222\|F\|0-13:A>T-13:A>T | | LOC105039218 | | XM_019848693.1 | XP_019704252.1 | P_GO:0009987 | cellular process | TGCAGATCTTTTCATTATTATCAGATACATTAGTTGAGATTCAAGAGCAAGCTTTGGATGATGATAATC | importin-9 isoform X1 | |
| Group 5 | 101271122\|F\|0-39:A>C-39:A>C | | LOC105042164 | | XM_019848832.2 | XP_019704391.1 | F_GO:0003676 | nucleic acid binding | TGCAGTGGCACATCTGCCACTCCCATCCTCCCCTCTGCCAGTGCACCTCCTCCTTGGAGGCTGGTTCAA | protein WRKY1 | |
| Group 5 | 101150085\|F\|0-10:C>T-10:C>T | | LOC105035317 | | XM_010910888.3 | XP_010909190.1 | F_GO:0005488 | binding | TGCAGTTCATCATACAAACAAAAAATTTACCTCAATTGATGTGGCAACTTTTCTTTCACTGATTTTTTC | casein kinase 1-like protein 2 | |
| Group 5 | 100889099\|F\|0-31:G>A-31:G>A | | LOC105045367 | | XM_010923624.3 | XP_010921926.1 | F_GO:0003677 | DNA binding | TGCAGTTCGAGACAACCCACTTGCTTCAGCCGCATCAATGAGATCCTGCCTTTTCATATAACTACTCCC | crossover junction endonuclease MUS81 | |
| Group 5 | 101253102\|F\|0-58:G>C-58:G>C | | LOC105050009 | | XM_019851804.2 | XP_019707363.1 | P_GO:0009987 | cellular process | TGCAGAGGGTCTTTTCTCTATCAAGTCTGATGTCTTTAGCTTTGGAGTCTTGCTTTTAGAGATCGGAAG | G-type lectin S-receptor-like serine/threonine-protein kinase At1g11300 | |
| Group 5 | 101109050\|F\|0-44:G>A-44:G>A | | LOC105040675 | | XM_010917427.3 | XP_010915729.1 | F_GO:0005515 | protein binding | TGCAGGTCCTGATGGATCACTAATTGGAATTCCAGGTAGTAGCTGTTCAAATTCTGAACTCTTTAGTTC | transcriptional corepressor LEUNIG isoform X2 | |
| Group 5 | 101145091\|F\|0-28:T>C-28:T>C | | LOC105047379 | | XM_010926293.3 | XP_010924595.1 | P_GO:0008150 | biological_process | TGCAGCTATTTAGGGGGTTAGCTTATATTCATGCTGGTCCAGGAGTTTGCCATAGGGATGTCAAGCCCC | shaggy-related protein kinase eta | |
| Group 5 | 100879349\|F\|0-55:C>T-55:C>T | | LOC105034025 | | XM_010909047.1 | XP_010907349.1 | F_GO:0005515 | protein binding | TGCAGCGAATTGGAGGTTTGAGATTTCGAATGGAACGTTTCAGGGGCACTATATTCCAAGCAGATTATT | chaperone protein dnaJ GFA2%2C mitochondrial | |
